# Targeted treatment and survival in advanced non-squamous non-small cell lung cancer patients – a nationwide and longitudinal study

**DOI:** 10.3389/fonc.2025.1506041

**Published:** 2025-02-20

**Authors:** Johanne Elise Nyen, Anja Ødegård Booth, Øyvind Husby, Christoffer Bugge, Ingrid Engebretsen, Francisco Oteiza, Åslaug Helland, Lars Fjellbirkeland, Odd Terje Brustugun, Bjørn Henning Grønberg

**Affiliations:** ^1^ Oslo Economics, Oslo, Norway; ^2^ Pfizer AS, Oslo, Norway; ^3^ Institute of Clinical Medicine, University of Oslo, Oslo, Norway; ^4^ Division of Cancer Medicine, Oslo University Hospital, Oslo, Norway; ^5^ Department of Respiratory Medicine, Oslo University Hospital, Oslo, Norway; ^6^ Section of Oncology, Drammen Hospital, Vestre Viken Hospital Trust, Drammen, Norway; ^7^ Department of Clinical and Molecular Medicine, Norwegian University of Science and Technology (NTNU), Trondheim, Norway; ^8^ Department of Oncology, St. Olavs Hospital, Trondheim University Hospital, Trondheim, Norway

**Keywords:** ROS, ALK, EGFR, real world data, lung cancer, time on treatment

## Abstract

**Objectives:**

We aimed to describe treatment patterns, time on treatment (ToT) and overall survival (OS) for patients with advanced non-squamous, EGFR+, ALK+ and ROS1+ NSCLC in Norway.

**Materials and methods:**

We extracted data on patients ≥ 18 years diagnosed with advanced non-squamous NSCLC between 2015 and 2022 from the Cancer Registry of Norway and data on cancer drug therapy from the Norwegian Patient Registry and the Norwegian Prescribed Drug Registry. ToT was measured from the date treatment was collected or administered until the last dispensing was depleted or last hospital drug administration. OS was measured from date of diagnosis until death.

**Results:**

In total, 5,279 patients were included, of whom 449 EGFR+, 131 ALK+ and 38 ROS1+. 75% of EGFR+ patients, 88% of ALK+ patients, and 58% of ROS1+ patients received at least one systemic treatment within the first three months after diagnosis. Median follow-up was 13, 19, and 4 months for EGFR+, ALK+, and ROS1+, respectively. The median ToT in first line (1L) for EGFR+ patients was 11 months for osimertinib (CI: 10.1-NA) and 9 months (CI: 8.2-11.2) for afatinib, dacomitinib, erlotinib and gefitinib. For ALK+ patients, median ToT in 1L was 20 months (CI: 14.7-23.7for alectinib, 11 months (CI: 4.7-NA) for brigatinib, and 7 months (CI: 2.9-21.6) for crizotinib. For the five ROS1+ patients treated with crizotinib in 1L, median ToT was 5 months (CI: 2.4-NA). For all patients with a targetable genomic alteration, unadjusted median OS was higher (p-value = 0.025) for patients diagnosed in 2020-2022 (median OS: 23 months, CI: 19.5-NA) compared to patients diagnosed in 2015-2019 (median: 19 months, CI: 16.5-21.2).

**Conclusions:**

ToT for targeted therapies was shorter than progression-free survival in clinical trials. However, patients eligible for targeted therapy still had a survival improvement during the study period.

## Introduction

Lung cancer accounts for about 13% of all new cancer diagnoses and is the leading cause of cancer-related deaths worldwide (1, 2). Approximately 80-85% of patients have non-small cell lung cancer (NSCLC) (3). About half of NSCLC patients are diagnosed with advanced, metastatic disease and have a poor prognosis (4). In Norway, 5-year relative survival for patients diagnosed with stage IV lung cancer was estimated to be 8.3% for patients diagnosed in the period 2018 through 2022 (3).

Over the past decades, several effective systemic therapies for NSCLC have been introduced (5, 6), largely because drugs designed to target oncogenic driver alterations have been developed (7). These protein kinase inhibitors (TKI’s) have proven to be more effective than chemotherapy regimens that used to be standard first line treatment for all advanced NSCLC. Since 2010, drugs targeting Epidermal Growth Factor Receptor (EGFR) mutations, Anaplastic Lymphoma Kinase (ALK) translocations and ROS1 fusions have become available through the Norwegian public health care system (EGFR-inhibitors since 2010, ALK-inhibitors since 2013 and ROS1-inhibitors since 2019). According to national guidelines, all non-squamous NSCLC tumors should be tested for EGFR-, ALK-, and ROS1-alterations since the corresponding inhibitors became available at public hospitals in our country (8). These three mutations are usually mutually exclusive and represents 11.5%, 2.4%, and 1-2% of adenocarcinomas, respectively (3, 9, 10).

The effectiveness of targeted therapies has also been seen in studies using real-world data (5, 11–14), but there is limited data on implementation rates of molecular testing, implementation of first-, second- and third-generation TKI’s, treatment across lines of therapy, ToT, and impact on overall survival (OS). Utilizing data from public Norwegian registries, we aimed to report such data for patients diagnosed with advanced non-squamous NSCLC in Norway between 2015 and 2022.

## Materials and methods

### Data sources

The study population was identified from the Cancer Registry of Norway (CRN). Data on drug treatment for each patient were collected from the CRN, the Norwegian Patient Registry (NPR) and the Norwegian Prescribed Drug Registry (NorPD).

Health institutions in Norway are required by law to notify CRN of any new cancer case, and CRN encompassed 99.2% of all lung cancer patients between 2018 and 2022 (15). Clinical stage (cTNM according to TNM v7 from 01.01.2015-31.12.2016, and v8 from 01.01.2017-31.12.2022) has been recorded for more than 80% of cases since 2017 (disease stage was classified as “local”, “regional” and “advanced” until 2017) (16). Data on EGFR and ALK status have been included in the CRN since 2013 and ROS1 status from 2022 onwards.

From the CRN, we extracted date of diagnosis, disease stage at diagnosis, histological subtype, biomarker (EGFR, ALK and ROS1) status, patient characteristics (sex, year of birth, and date of death if applicable), and whether patients underwent surgery or radiation therapy.

Data on medical treatment were collected from multiple sources. The NorPD include data on all subcutaneous and oral cancer drugs dispensed at Norwegian pharmacies from 2004, the NPR holds information on all hospital encounters (in- and outpatient visits) and hospital administered drugs from 2008, and the CRN holds information on hospital administered drugs from 2008 and all cancer drugs administered subcutaneously and oral from 2019. The CRN does not cover drug treatments in hospitals in the Northern region (approx. 10% of the population), but all Norwegian hospitals are covered by the NPR. Combined, these data hold information on all medical systemic treatment administered at public hospitals during the study period except oral study drugs dispensed through clinical trials.

### Study population

We extracted data on all patients aged 18 or above diagnosed with advanced non-squamous NSCLC (stages IIIB, IIIC or IV) between January 1, 2015, and December 31, 2022 according to the CRN. Patients who were diagnosed with lower stage disease and later developed advanced disease were not included, and we excluded patients treated with curative intent in the primary setting.

We then defined three biomarker-defined subgroups (EGFR+, ALK+ and ROS1+) and one with the remaining non-squamous NSCLC patients. Patients were assigned to subgroups if they a) were registered as being biomarker positive in the CRN or b) received a specific targeted treatment within the first three months of diagnosis. The time period for inclusion of patients to biomarker subgroups were based on when targeted therapies were approved for use in the public health care sector and when biomarker results were reported to CRN.

#### EGFR+

Patients registered as being EGFR+ in the CRN (n = 431) and patients with unknown EGFR-status who received an EGFR inhibitor (afatinib, dacomitinib, gefitinib or osimertinib) within the first three months since diagnosis (n = 18). Erlotinib-treatment was not used to assign patients to this group since it is sometimes used to treat patients who are not EGFR+ (30 patients without known EGFR+ received erlotinib within the first three months of diagnosis). One patient was recorded as being both EGFR+ and ALK+, while eight patients were both EGFR+ and ROS1+. These patients were assigned to the EGFR+ subgroup since they received EGFR-inhibitor therapy.

#### ALK+

Patients recorded as being ALK+ in the CRN (n = 119) and patients with unknown ALK-status who received an ALK inhibitor (alectinib, brigatinib or ceritinib) within the first three months of diagnosis (n = 12). Lorlatinib-treatment was not used to assign patients to the ALK+ subgroup as lorlatinib was not recommended for first line treatment during the study period. Patients treated with crizotinib were included if they received alectinib or brigatinib as subsequent treatment, since these are likely to have been considered to have ALK+ and not ROS1+ disease.

#### ROS1+

Patients recorded as being ROS1+ in the CRN (n = 36) and patients with unknown ROS1-status who received entrectinib after crizotinib treatment (n = 2), since these are likely to have been considered having ROS1+ and not ALK+ disease.

#### Other non-squamous NSCLC

All other non-squamous NSCLC patients in the study population.

### Variables and outcomes

#### Treatment classification

Systemic drug treatment was identified based on the Anatomical Therapeutic Chemical (ATC) code and classified as protein kinase inhibitors (targeted therapy), chemotherapy (ChT), or immunotherapy (IO), according to CRNs classification (17, 18).

Erlotinib (L01EB02), afatinib (L01EB03), and gefitinib (L01EB01) were all as first line treatment options for EGFR+ patients before 2013, while dacomitinib (L01EB07) and osimertinib (L01EB04) were introduced as first line treatments with public reimbursement in 2020 and 2021, respectively.

For ALK+ patients, crizotinib (ATC-code L01XE16) was approved for use in the public health care sector in Norway as second line treatment in 2012, and as first line treatment in 2017. Alectinib (L01ED03) and ceritinib (L01ED02) were approved as first line treatment in 2018. Brigatinib (L01ED04) was approved as second line treatment following crizotinib in 2019, and as a first line treatment in 2021. Lorlatinib (L01ED05) was approved as second line treatment for ALK+ patients in 2019 and as a first line treatment in 2022.

For ROS1+ patients, crizotinib (L01XE16) was approved as first line treatment in 2018 and entrectinib (L01EX14) in 2021.

Quadruple treatment was defined as combination treatment with atezolizumab (L01FF05), bevacizumab (L01FG01), paclitaxel (L01CD01) and carboplatin (L01XA02). Platinum doublet treatment was defined as treatment with cisplatin (L01XA01) or carboplatin (L01XA02) in combination with vinorelbine (L01CA04), etoposide (L01CB01), paclitaxel (L01CD01), pemetrexed (L01BA04) or gemcitabine (L01BC05).

#### Treatment patterns

As clinicians may prescribe IO and/or ChT while they wait for biomarker test results, first line treatment was defined as the first targeted therapy received within three months since diagnosis. If no targeted therapy was given during the first three months, the first non-targeted therapy (received within three months) was considered first line treatment. Three months is deemed as a reasonable threshold after which biomarker test results should have been received and acted upon by clinicians.

Treatment patterns were presented using Sankey flow diagrams, a data visualization technique that allows for describing change of treatment across treatment lines (19). Line not reached (LNR) indicates that patients were still on treatment at the end of the study period (last 12 weeks of the data collection period).

#### Time on treatment

ToT was estimated using the Kaplan-Meier estimator (20) and presented as drug survival curves and median ToT (mToT). ToT was estimated based on the defined daily dose (DDD) for each drug dispensing (targeted therapies) or assumed to be four weeks on average per treatment course for IO and ChT. ToT was estimated for first line treatments and for all treatment lines combined (i.e., total ToT for all treatment lines (mTToT), allowing for drug switch). Drug treatment was considered discontinued when a) patients did not receive a new drug after the previous one would have been depleted, b) a treatment gap of 12 weeks or more, c) death, or if another drug treatment was administered.

#### Overall survival

OS was estimated from date of diagnosis to death or end of follow-up using the Kaplan-Meier estimator (20). To investigate changes in OS over time, results were stratified based on year of diagnosis (2016-2019 vs 2020-2022 for ALK+ and 2015-2019 vs 2020-2022 for all other subgroups).

### Reporting guidelines and ethics

This study follows Strengthening the Reporting of Observational Studies in Epidemiology (STROBE) reporting guideline for observational studies. The study was approved by the Regional Ethics Committee of Norway South-East D (Reference number 485084) and registered at http://ClinicalTrials.gov (Reference number NCT05834348). All analyses were conducted using R version 4.1.2 (2021).

## Results

### Patient characteristics

**Figure 1:**
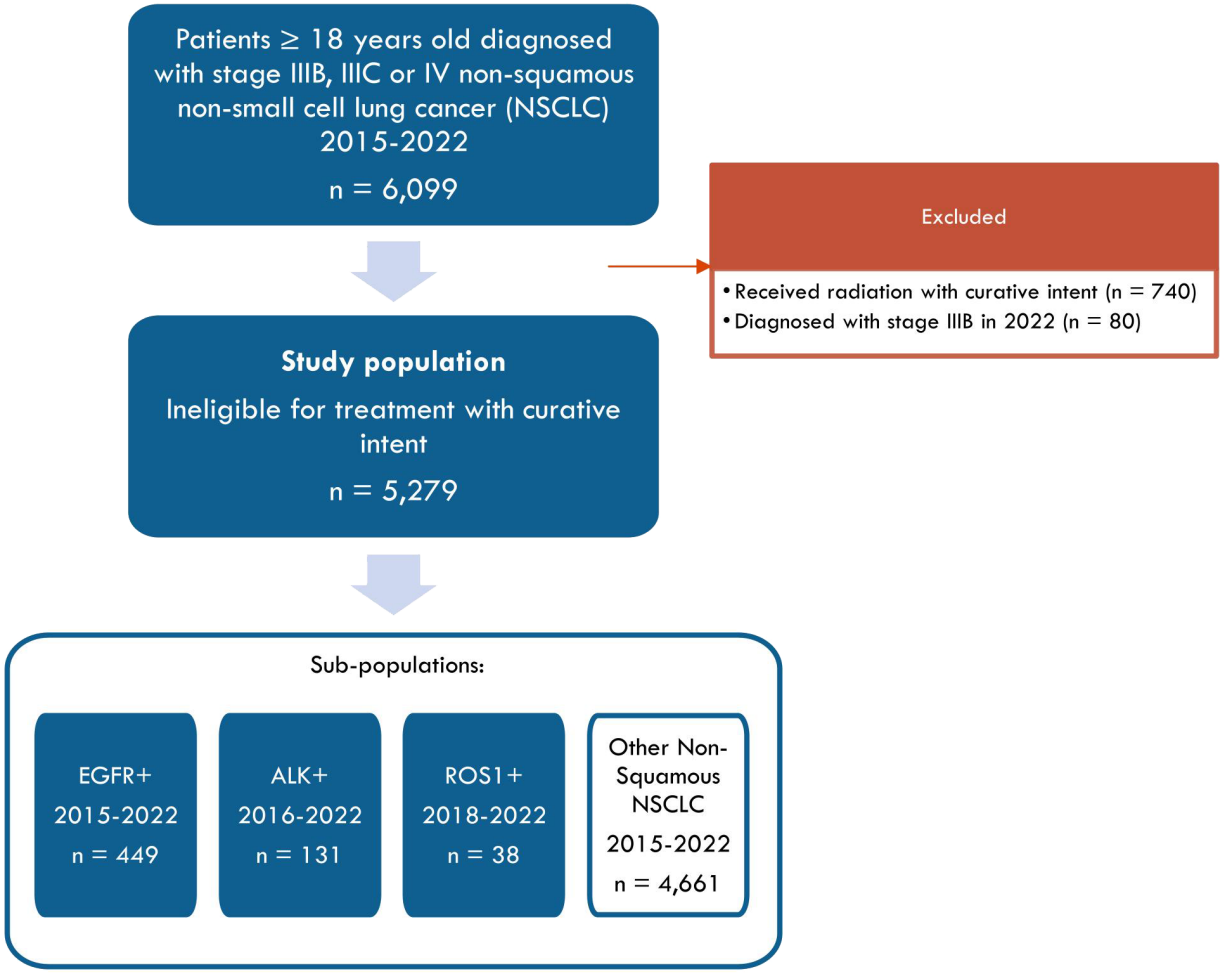
Study flow chart.

The overall population comprised 5,279 patients (**Figure 1**). Baseline characteristics are listed in **Table 1**. Median age was 71 (25^th^ and 75^th^ percentile: 64, 77) years, and 48% were female. Median follow-up was 5.5 (25^th^ and 75^th^ percentile: 1.9, 14.1) months.

**Table 1:** Patient characteristics for patients diagnosed with advanced NSCLC in Norway between 2015 and 2022.

	All	All biomarker subgroups	EGFR+	ALK+	ROS1+	Other non-squamous NSCLC
Number of patients	5279	618	449	131	38	4661
Year of diagnosis	2015-2022	2015-2022	2015-2022	2016-2022	2018-2022	2015-2022
Median age at diagnosis (25^th^ percentile, 75^th^ percentile)	71 (64, 77)	69 (59, 77)	70 (59, 78)	64 (52.5, 73)	74.5 (62.5, 78)	71 (65, 77)
Mean age at diagnosis (SD)	70.2 (10.2)	67.1 (13)	68.3 (12.6)	62.6 (13.3)	69.5 (13.3)	70.7 (9.7)
Proportion female	48.3%	62.9%	65.7%	51.9%	68.4%	46.3%
Percent received targeted therapy	10.3%	87.4%	88.3%	94.1%	46.4%	1.8%
Median follow-up (from diagnosis until death/end of data) (months) (25^th^ percentile, 75^th^ percentile)	5.5 (1.9, 14.1)	13.4 (4.6, 24.5)	12.7 (5.3, 24)	18.7 (5.8, 32.7)	4.0 (1.6, 8.9)	4.9 (1.6, 12.5)
Percent dead during study period	78.5%	58.9%	63.7%	47.3%	42.1%	81.1%
Stage at time of diagnosis						
IIIB	5.4%	3.5%	3.6%	4.6%	0%	5.6%
IIIC	2.9%	1.6%	1.1%	1.5%	7.9%	3.1%
IVA	36.2%	36.2%	36.1%	35.1%	42.1%	36.2%
IVB	55.5%	58.6%	59.2%	58.8%	50.0%	55.1%
Morphology						
Adenocarcinoma	82.4%	94.7%	95.3%	92.4%	94.7%	80.8%
Non-small cell carcinoma UNS**	15.3%	5.0%	4.6%	6.1%	5.3%	16.7%
Large cell neuroendocrine carcinoma	2.2%	0.3%	0%	1.5%	0%	2.4%

SD, Standard deviation.

618 patients were assigned to one of the three biomarker subgroups (EGFR+, ALK+, or ROS1+). In general, ALK+ patients (median age 64, 25^th^ and 75^th^ percentile: 52.5, 73) were younger than EGFR+ (median 70 years,25^th^ and 75^th^ percentile: 59, 78) and ROS1+patients (median 74.5 years,25^th^ and 75^th^ percentile: 62.5, 78), and the proportion of females was lower (ALK+ 52%, EGFR+ 66%, ROS1 + 68%). There were no differences in stage distribution or proportions with adenocarcinomas across these subgroups.

Median follow-up was 12.7 months (25^th^ and 75^th^ percentile: 5.3, 24) for EGFR+ patients, 18.7 months (25^th^ and 75^th^ percentile: 5.8, 8.9) for ALK+ patients, and 4.0 months (25^th^ and 75^th^ percentile: 1.6, 8.9) for ROS1+ patients. During follow-up, 88% of EGFR+, 94% of ALK+, and 46% of ROS1+ patients received at least one targeted therapy.

For other non-squamous NSCLC patients, median age at diagnosis was 71 years (25^th^ and 75^th^ percentile: 65, 77), proportion females was 46%, median follow-up time was 4.9 months (25^th^ and 75^th^ percentile: 1.6, 12.5), and 2% received a targeted therapy during the study period.

### Treatment patterns and time on treatment

#### EGFR+

**Figure 2:**
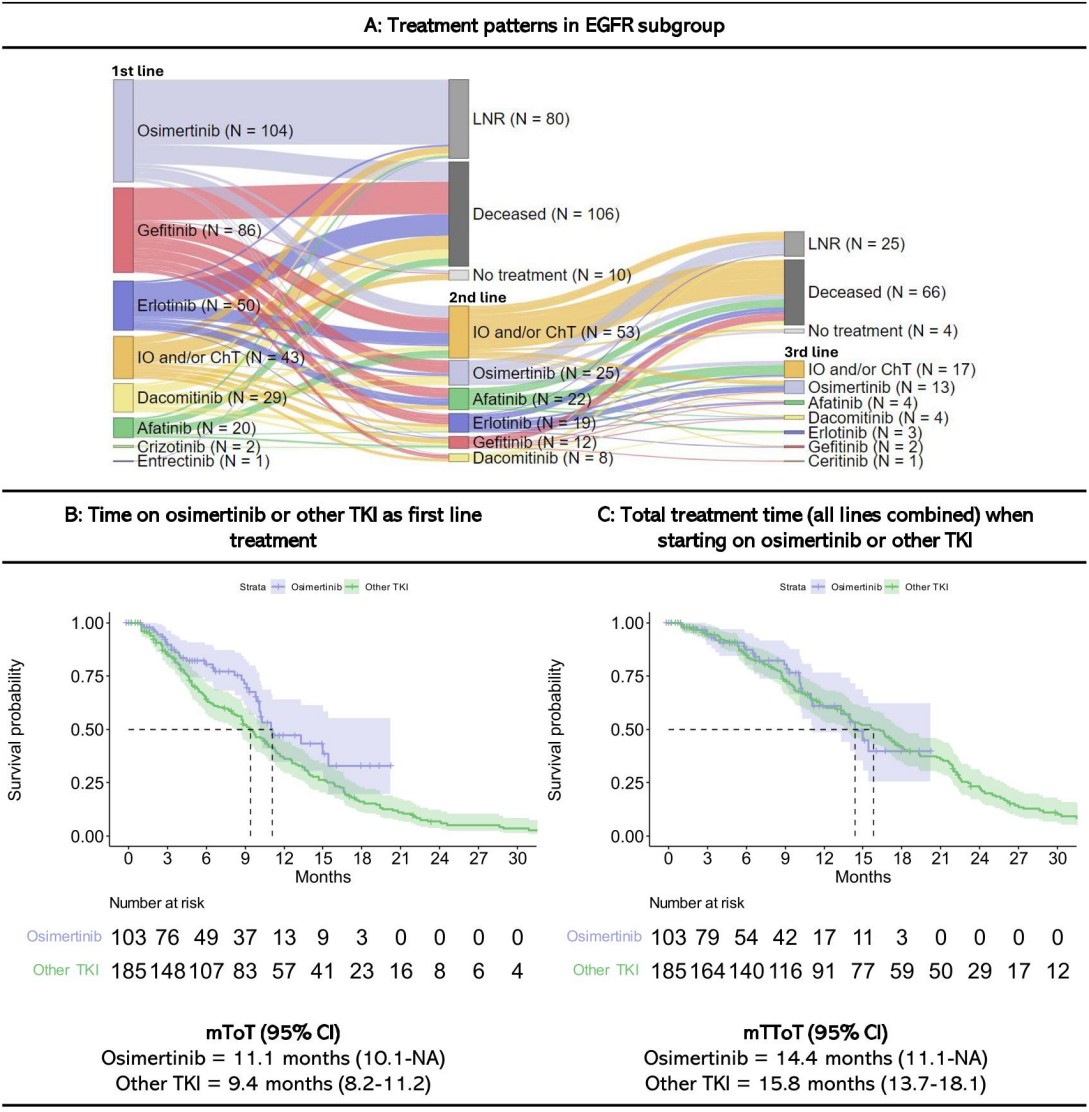
Treatment patterns and time on treatment for EGFR+ patients starting treatment within three months since diagnosis. Figures are restricted to EGFR patients who received treatment within the first three months since diagnosis. 114 patients neverreceived systematic treatment within the first three months since diagnosis. A detailed description of treatment patterns by patient is provided in **Supplementary Figure 4**. IO, Immunotherapy; ChT, Chemotherapy; LNR, Line not reached; Deceased, Dead prior to reaching line; mToT, median time on treatment; mTToT, median total time on treatment; TKI, Tyrosine Kinase Inhibitors (other TKI includes afatinib, dacomitinib, erlotinib and gefitinib); Cl, confidence interval. **(A)** Treatment patterns, **(B)** Time on osimertinib or other TKI in first line, and **(C)** Total treatment time (all lines combined) when starting on osimertinib or other TKI in EGFR+ patients.

449 patients were categorized as EGFR+ patients, of which 75% (n=335) received systemic treatment outside of clinical studies within the three first months after diagnosis (**Figure 2**). Overall, osimertinib was the most common first line treatment (31% of those who received first line treatment, n=104), followed by gefitinib (26%, n=86) and erlotinib (15%, n=50). The choice of first line EGFR-inhibitor therapy changed during the study period according to changes in national guidelines and time of reimbursement (8). Afatinib, erlotinib and gefitinib were most commonly used prior to 2020, while osimertinib was most commonly used after reimbursement for first line therapy was approved in 2021.

Of the 335 EGFR+ patients who received first line treatment, 41% (n=139) received second-line treatment, most commonly IO and/or ChT (38% of those who received second line treatment, n=53), osimertinib (18%, n=25), afatinib (16%, n=22), and erlotinib (14%, n=19). Of the 196 patients who did not receive second-line treatment, 41% (n=80) were still on first line treatment at the end of follow-up (LNR), 54% (n=106) died while on first line treatment, and 5% (n=10) stopped treatment after first line but were alive at the end of the study period (follow-up of 14 to 88 weeks without treatment).

Among the 139 patients who received second-line treatment, 32% patients (n=44) continued to third line, most commonly IO and/or ChT (39% of those who received third line treatment, n=17) or osimertinib (30%, n=13).

Swimmer plots showing the length of treatment duration for each EGFR+ patient is presented in **Supplementary Figures 4A1, A2**.

Patients who received osimertinib in first line had a mToT of 11 months on osimertinib (CI: 10.1-NA), and a mTToT of 14 months (CI: 11.1-NA) for all lines. The mToT for the first line treatment with the other EGFR-inhibitors (afatinib, dacomitinib, erlotinib and gefitinib) was 9.4 months (CI: 8.2-11.2), and total mTToT was 15.8 months (CI: 13.7-18.1). Results for each individual treatment are presented in **Supplementary Figure 3**.

In total, 29 EGFR+ patients were treated with platinum doublet while 19 patients received quadruple treatment after targeted therapy. The mToT on these treatments were 3.0 and 2.6 months, respectively (**Supplementary Figure 2**).

#### ALK+

**Figure 3:**
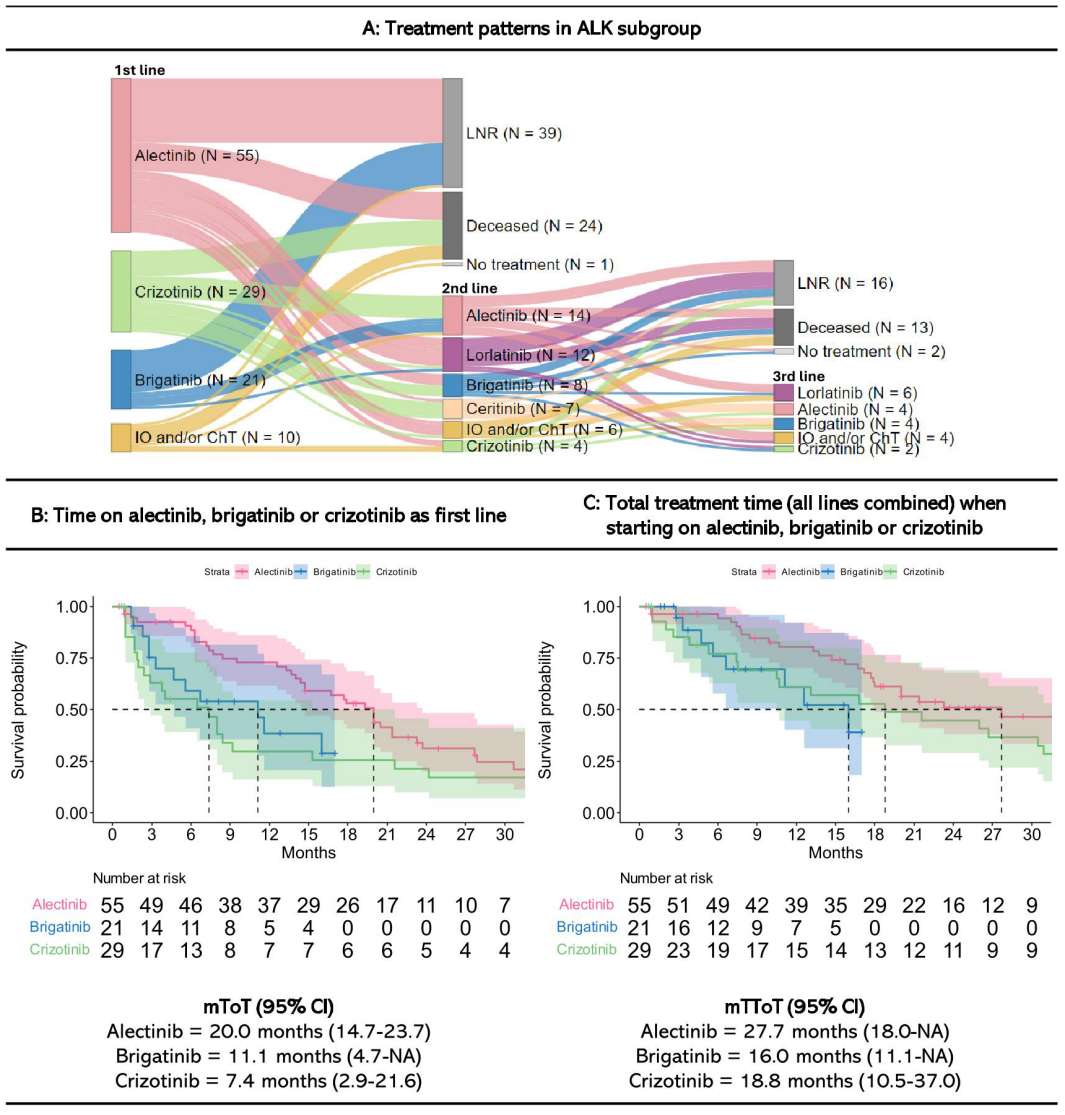
Treatment patterns and time on treatment for ALK+ patients starting treatment within three months since diagnosis. Figures are restricted to patients who received treatment within the first three months since diagnosis. 16 patients never received systematic treatment within the first three months since diagnosis. A detailed description of treatment patterns by patient is provided in **Supplementary Figure 4**. IO, Immunotherapy; ChT, Chemotherapy; LNR, Line not reached; Deceased, Dead prior to reaching line; NA, Not annotated; mToT, median time on treatment; mTToT, median total time on treatment; Cl, confidence interval. **(A)** Treatment patterns, **(B)** Time on alectinib, brigatinib, and crizotinib as first line, and **(C)** Total treatment time (all lines combined) when starting on alectinib, brigatinib or crizotinib in the ALK+ subgroup.

There were 131 patients defined as having ALK+ disease. Among these, 88% patients (n=115) received systemic treatment within the first three months since diagnosis (**Figure 3**). The most common first line treatments were alectinib (48% of those who received first line treatment, n=55), crizotinib (25%, n=29), and brigatinib (18%, n=21).

Of the 115 patients who received first line treatment, 44% patients (n=51) received second line treatment, most commonly alectinib (26%, n=14) or lorlatinib (22%, n=12). Among the remaining 64 patients who did not receive second line treatment, 61% patients (n=39) were still on first line treatment at the end of follow-up (LNR). Furthermore, 38% (n=24) died before reaching a subsequent treatment line. One patient stopped treatment after 27 months of first line treatment, but was still alive at the end of follow-up after 11 months without treatment.

Of the 51 patients who received a second line treatment, 39% (n=20) reached a third line, most commonly lorlatinib (30% of those who received a third line treatment, n=6). Among the remaining 31 patients who did not receive third line treatment, 52% patients (n=16) were still on second line treatment with alectinib, lorlatinib, ceritinib or crizotinib at the end of follow-up (LNR), whereas 42% (n=13) died while on second line treatment. Two patients stopped treatment but were still alive at the end of follow-up.

Swimmer plots showing the length of treatment duration for each ALK+ patient is presented in **Supplementary Figure 4B**.

Patients treated with alectinib in first line had a mToT on alectinib of 20 months (CI: 14.7-23.7). When combining all lines of treatment, the mTToT was 28 months (CI: 18-NA) (i.e., median time on subsequent treatment was 8 months). Those who received lorlatinib had the longest time on second-line treatment (median of 13 months, CI: 5.3-NA) (**Supplementary Figure 1**).

Patients treated with brigatinib in first line had a mToT of 11 months (CI: 4.7-NA), while their mTToT for all lines of treatment was 16 months (CI: 11.1-NA). For first line crizotinib, corresponding numbers were 7 (CI: 2.9-21.6) and 19 months (CI: 10.5-37.0).

#### ROS1+

**Figure 4:**
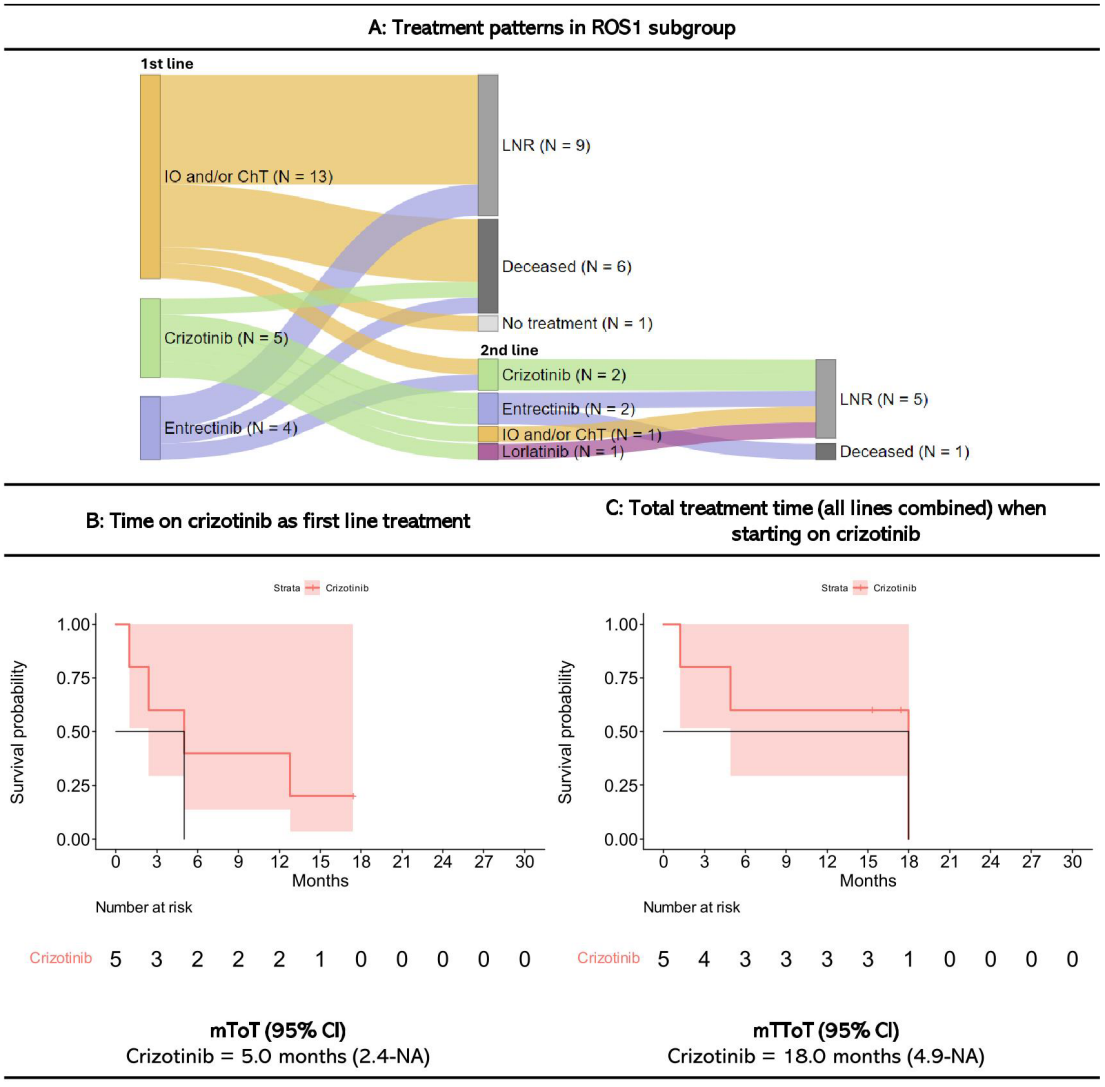
Treatment patterns and time on treatment for ROS1+ patients starting treatment within three months since diagnosis. Figures are restricted to ROS1 patients who received treatment within the first three months since diagnosis. 16 patients never received systematic treatment within the first three months since diagnosis. A detailed description of treatment patterns by patient is provided in **Supplementary Figure 4**. IO, Immunotherapy; ChT, Chemotherapy; LNR, Line not reached; Deceased, Dead prior to reaching line; mToT, median time on treatment; mTToT, median total time on treatment; Cl, confidence interval. **(A)** Treatment patterns, **(B)** Time on crizotinib as first line treatment, and **(C)** Total treatment time (all lines combined) when starting on crizotinib for ROS1+ patients.

38 patients were assigned to the ROS1+ subgroup, of whom 58% (n=22) received systemic treatment within three months since diagnosis (**Figure 4**). Even though targeted therapy was available, the most common first line treatment was IO and/or ChT (59% of those who received first line treatment, n=13). Of the patients receiving first line treatment, 31% (n=7) never received second line treatment, while 41% (n=9) were still on first line treatment at the end of follow-up on.

Swimmer plots showing the length of treatment duration for each ROS1+ patient is presented in **Supplementary Figure 4C**.

The five patients treated with crizotinib as first line therapy had a mToT of 5 months (CI: 2.4-NA), and a mTToT of 18 months (CI: 4.9-NA) (i.e., the treatment given post crizotinib resulted in 13 more months on treatment).

### Overall survival

**Figure 5:**
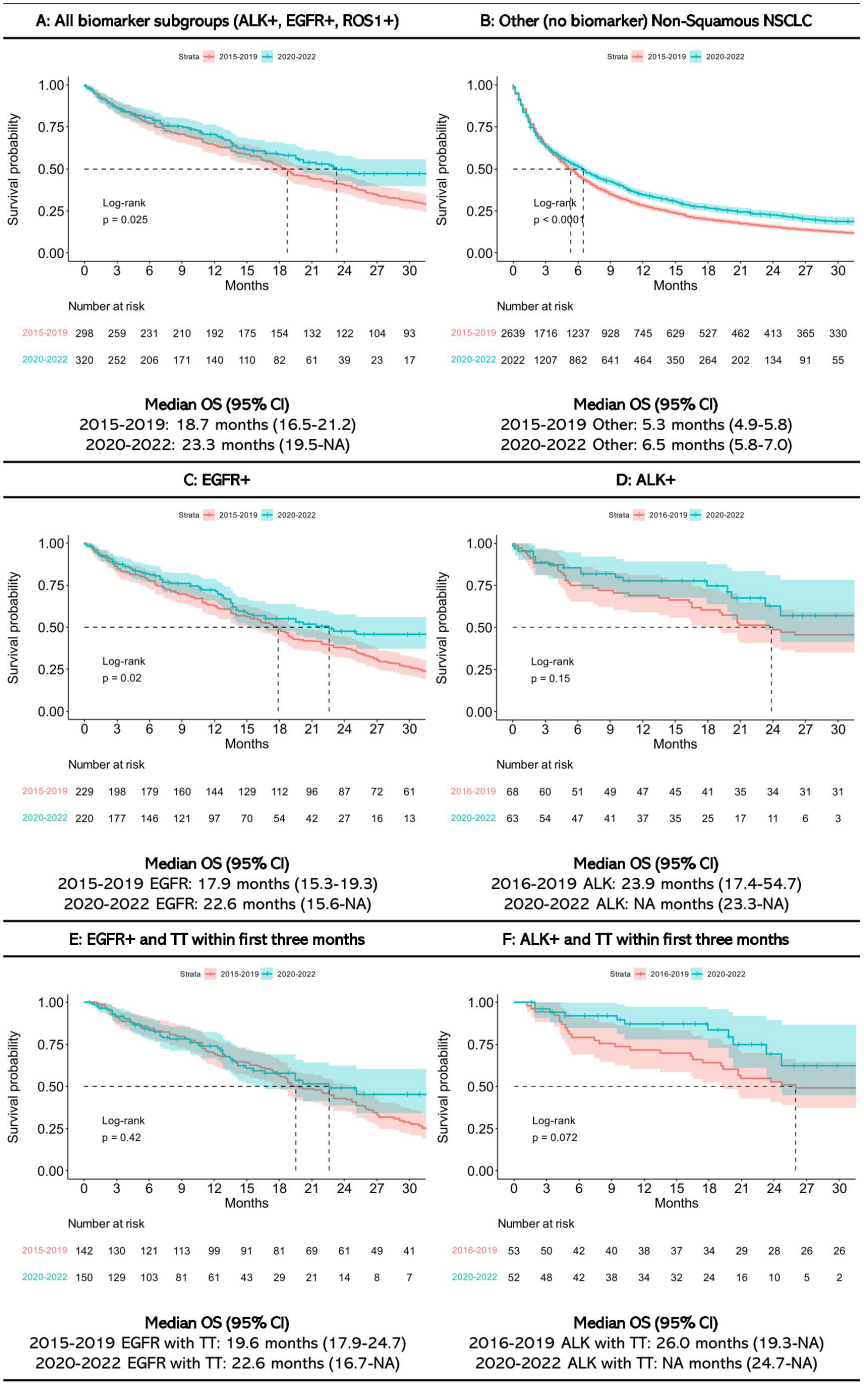
Overall survival in **(A)** All biomarker subgroups, **(B)** Other Non-Squamous NSCLC, **(C)** EGFR+, **(D)** ALK+, **(E)** EGFR+ and targeted treatment (TT) within first three months, and **(F)** ALK+ and TT within first three months.

For all patients assigned to biomarker subgroups, median OS was 19 months (CI: 16.5-21.2) for those diagnosed between 2015 and 2019, and 23 months (CI: 19.5-NA) for those diagnosed between 2020 and 2022 (**Figure 5**). Median OS among EGFR+ patients was 18 months (CI: 15.3-19.3) and 23 months (CI: 15.6-NA) for those diagnosed between 2015-2019 and 2020-2022, respectively. Median OS among ALK+ patients diagnosed in the earlier years was 24 months (CI: 17.4-54.7), and not reached for those diagnosed between 2020 and 2022 (CI: 23.3-NA). OS for ROS1+ patients was not estimated due to small sample size. Other patients (no biomarker) with non-squamous NSCLC had a median OS of 5 months (CI: 4.9-5.8) (2015-2019) and 7 months (CI: 5.8-7.0) (2020-2022). 1-year and 2-year overall survival rates are presented in **Supplementary Table 3**.

## Discussion

Most patients diagnosed with advanced non-squamous NSCLC with a confirmed biomarker for EGFR, ALK or ROS1 in Norway from 2015 to 2022 received systemic treatment within the first three months since diagnosis (75%, 88%, and 58%, respectively). For EGFR+ patients, the mToT was 11.1 months osimertinib in first line, compared to 9.4 months for the other EGFR-inhibitors. ToT on platinum doublet or quadruple treatment following targeted therapy was limited and similar for both regimens. Among those who received an ALK-inhibitor in the first line, mToT in first line were longer for alectinib (20.0 months) compared to brigatinib (11.1 months) and crizotinib (7.4 months). For all patient subgroups, the mOS was higher for patients diagnosed in 2020-2022 compared to patients diagnosed in 2015-2019, but the survival improvement was larger for patients receiving targeted therapies than for other patients.

Our CRN data does not contain information on progression dates, and response evaluations are not always done as stringent in clinical practice as in trials. However, the ToT we observed may serve as an indirect measure of progression-free survival (PFS) (21). In ARCHER1050, the authors report a mPFS of 14.7 months for dacomitinib and 9.2 months for gefitinib for EGFR+ patients (22). The results are somewhat higher than our estimated mToT (8.2 months for dacomitinib and 8.9 months for gefitinib). In the FLAURA trial, the authors found a mPFS of 18.9 months for osimertinib and 10.2 months for patients treated with erlotinib or gefitinib (23). In our data, mToT on osimertinib was 11.1 months while the other EGFR inhibitors had 2-3 months shorter mToT. The ALEX study (24) reported a mPFS of 34.8 months for alectinib and 10.9 months for crizotinib, while it was 24.7 months for brigatinib and 9.4 months for crizotinib (results from the non-Asian population) in the ALTA 1L study (25). Similar results have also been reported by researchers using real-world data (26, 27). In comparison, we found a mToT of 20.0 months (alectinib), 11.1 months (brigatinib), and 7.4 months (crizotinib), which corresponds to findings from a population-based study from Denmark (12). A North American study investigating time on lorlatinib as second line treatment for ALK+ patients (28) found comparable results to our study, with mToT of 15.3 months for lorlatinib in second line. In the PROFILE 1001 (29), the reported mPFS for crizotinib in ROS1+ patients was 19.3 months, compared to a mToT of 5 months in our study. Our estimated mToT is also lower than findings from previous real world evidence studies. A sytematic litterature review and meta-analysis found a mPFS 14.5 months for crizotinib in ROS1+ patients which is more in line with the results from PROFILE 1001 (30). The patients in our data are older (median age is 74) than in PROFILE 1001 (median age 55) and in the sytematic litterature review where the median age ranged from 48-68. The follow-up time and sample size for ROS1+ patients in our study were limited due to the relative recent introduction of ROS1+testing in Norway.

Shorter ToT observed in clinical practice than in randomized controlled trials may have several explanations. Participants in clinical trials are in general younger, have better performance status, less comorbidity, and are usually followed more closely than most patients seen in the clinic. In addition, we excluded patients who develop metastases after receiving potentially curative treatment. These patients may have a better prognosis than those diagnosed with *de novo* advanced disease (31).

In our study, the median OS for EGFR+ patients increased from 18 months for those diagnosed in 2015-2019 to 23 months for those diagnosed in 2020-2022. The ARCHER1050 study reported a mOS of 34.1 months in the dacomitinib arm, and 27.0 months in the gefitinib arm (30), while the FLAURA study reported a mOS of 38.6 months for patients on osimertinib and 31.8 months among those receiving erlotinib or gefitinib (32). The median 1-year OS increased from 69% (95% CI: 59-81%) for ALK+ patients diagnosed in 2016-2019 to 78% (95% CI: 68-89%) for those diagnosed in 2020-2022. In the ALEX study, the 1-year OS for patients in the alectinib arm was 84%, while it was 83% for those in the crizotinib arm (24). Treatment switches, a more heterogenous population (as discussed above regarding ToT) may explain the survival differences between these trials and our study cohort. For example, a study concluded that patients treated with osimertinib in first line who were ineligible for the FLAURA-trial had 18 months shorter median OS than those who were eligible for that trial (33). Although our study does not enable us to assess a potential causal relationship between the introduction of targeted therapies in advanced NSCLC and increased OS, we did, in line with a previous study (5), observe an OS improvement after the introduction of targeted therapies in general, and with the introduction of later generation agents.

The main strength of this study lies in the completeness of our CRN which covers 99.2% of all lung cancer patients in Norway. Furthermore, our health care services are public, and access to services is regarded independent of income, societal status, age, etc., although some differences are unveiled (34). Thus, the national registries cover virtually all Norwegian NSCLC cancer patients and much information about the treatment they receive. National treatment guidelines are well recognized by the clinical communities and are believed to ensure quite uniform treatment across hospitals and regions. However, the study has several limitations. First, we did not have information on oral drug treatment received by participants in clinical trials (e.g., the TREM-study (35) which offered second-line osimertinib (enrolment period 2015-2017) or the ongoing FIOL-study which offered first-line osimertinib therapy to EGFR+ patients (enrolment period 2018-2022) (36)), which probably explains why a lower proportion of EGFR patients (88.3%) in our cohort were recorded to have received targeted therapy than ALK+ patients (94.1%). Second, methods for molecular testing vary between hospitals, but our Cancer Registry do not include information on the methods used. Third, although ROS1 testing was implemented in 2019, the results were not reported to CRN prior to 2022. Fourth, EGFR/ALK status was missing in the CRN for 30-36% of the patients between 2017 and 2022. Test rates for EGFR and ALK increased during the study period from 75% to 85% for EGFR, and from 70% to 89% for ALK patients (37, 38). Thus, the assignment to subgroups was made based on the treatment received for 4.0% of the EGFR+ patients (we exclude erlotinib-treatment for allocation to this group, but this accounted for only 30 patients and is not likely to have influenced our results), 9.2% of the ALK+ patients, and 5.3% for ROS+ patients. Although having a confirmed test result is preferable, we consider it unlikely that patients have received these specific targeted therapies without having the relevant oncogenic alteration. Fifth, we only had data on drugs dispensed to patients and DDD, not prescribed doses. Some patients may have used a higher or lower dose than the DDD, which may influence the estimated ToT. Several factors may determine the choice of drug treatment (e.g., clinician or patient preferences) and whether the patient discontinue treatment. Most importantly, our CRN does not contain information on whether treatments were discontinued due to toxicity, whether treatment was continued beyond progression, and we did not assess whether e.g. chemotherapy was added to targeted therapy. This hampers interpretation of the data on treatment beyond the first line. Most notably, the quadruple combination does not appear to provide any clinical benefit over chemotherapy alone, but the numbers are small. Finally, since the different drugs became available at different timepoints, the observation period varies, which might explain why the total ToT did not increase with the introduction of osimertinib as first-line treatment of EGFR+ patients, whereas the survival time did improve.

The treatment landscape for advanced NSCLC has changed rapidly over the last years, and studies like ours can serve as important evaluations of to what extent changes in diagnostic workup, especially molecular testing, and treatment have been implemented. During the study period, only targeted therapies for EGFR+, ALK+, and ROS1+ NSCLC were available at public hospitals in Norway, and these were the subgroups with sufficient follow-up data to include in this study. Currently, more targeted therapies are available and NSCLC tumors are now being tested for a broader range of oncogenic drivers. Furthermore, reports like ours serve as valuable supplements to results from randomized controlled trials on selected patients which inform both clinicians, patients, relatives and decision makers in health care about the clinical impact of new therapies. Considering that high costs of new cancer drugs have become a challenge for most health services, such data might also be used to support both primary and *post-hoc* evaluations of cost-effectiveness of drugs. Economic evaluations are commonly based on data from trials, including participants that do not necessarily represent the typical patients seen in the clinic. Registry data as presented in this report may provide valuable information to decision makers when seen in combination with the results from trials.

### Conclusion

The vast majority of Norwegian advanced non-squamous NSCLC patients with targetable oncogenic alterations receive appropriate targeted therapy, and these patients have a much longer survival time than patients without such alterations, confirming the effectiveness of these therapies in patients seen in everyday clinical practice. There was an encouraging survival improvement during the study period which may be attributed to the introduction of later generation agents, though the observed mToT for the targeted therapies was shorter than reported mPFS in clinical trials.

## Data Availability

According to Norwegian legislation, the Norwegian Data Protection Authority and the Norwegian Directorate of eHealth, we are not allowed to share original study data publicly. Requests to access these datasets should be directed to helsedata.no, service@helsedata.no.

## References

[1] Sung H Ferlay J Siegel RL Laversanne M Soerjomataram I Jemal A. Global cancer statistics 2020: GLOBOCAN estimates of incidence and mortality worldwide for 36 cancers in 185 countries. CA: A Cancer J Clin. (2021) 71:209–49. doi: 10.3322/caac.21660 33538338

[2] Ferlay J Colombet M Soerjomataram I Parkin DM Piñeros M Znaor A. Cancer statistics for the year 2020: An overview. Int J Cancer. (2021) 149:778–89. doi: 10.1002/ijc.v149.4 33818764

[3] Kreftregisteret. Årsrapport 2023 med resultater og forbedringstiltak fra Nasjonalt kvalitetsregister for lungekreft. Oslo: Kreftregisteret (2024).

[4] Bauman JR Edelman MJ. Targeted therapies in non-small cell lung cancer. In: Jeremić B, editor. Advances in Radiation Oncology in Lung Cancer. Springer International Publishing, Cham (2023). p. 347–69.

[5] Børø S Thoresen S Helland Å. Improvements in survival for patients with stage IV adenocarcinoma in the lung, diagnosed between 2010 - 2020 - A population-based registry study from Norway. Front Oncol. (2022) 12:1017902. doi: 10.3389/fonc.2022.1017902 36523970 PMC9745181

[6] Brustugun OT Grønberg BH Fjellbirkeland L Helbekkmo N Aanerud M Grimsrud TK. Substantial nation-wide improvement in lung cancer relative survival in Norway from 2000 to 2016. Lung Cancer. (2018) 122:138–45. doi: 10.1016/j.lungcan.2018.06.003 30032822

[7] Araghi M Mannani R Maleki AH Hamidi A Rostami S Safa SH. Recent advances in non-small cell lung cancer targeted therapy; an update review. Cancer Cell Int. (2023) 23:162. doi: 10.1186/s12935-023-02990-y 37568193 PMC10416536

[8] Helsedirektoratet. Ikke-kurativ behandling av ikke-småcellet lungekreft: Målrettet behandling (2024). Available online at: https://www.helsedirektoratet.no/retningslinjer/lungekreft-mesoteliom-og-thymom-handlingsprogram/ikke-kurativ-behandling-av-ikke-smacellet-lungekreft/malrettet-behandling (Accessed June 1, 2024).

[9] Kreftregisteret. Årsrapport 2022 med resultater og forbedringstiltak fra Nasjonalt kvalitetsregister for lungekreft. Oslo: Kreftregisteret (2023).

[10] Flodgren G Hamidi V. Tests for detection of ROS1 gene alterations in people with non-small cell lung cancer (NSCLC): A Health Technology Assessment. Oslo: Norwegian Institute of Public Health (2021).

[11] Helland Å Myklebust TÅ Conte S Frederiksen LE Aarøe J Enerly E. EGFR-mutation testing, treatment patterns and clinical outcomes in patients with stage IB-IIIA non-small cell lung cancer in Norway-a nationwide cohort study. Cancer Treat Res Commun. (2024) 38:100785. doi: 10.1016/j.ctarc.2023.100785 38190787

[12] Hansen KH Johansen JS Urbanska EM Meldgaard P Hjorth-Hansen P Kristiansen C. Clinical outcomes of ALK+ non-small cell lung cancer in Denmark. Acta Oncol. (2023) 62:1775–83. doi: 10.1080/0284186X.2023.2263153 37815923

[13] Marin-Acevedo JA Pellini B Kimbrough EMO Hicks JK Chiappori A. Treatment strategies for non-small cell lung cancer with common EGFR mutations: A review of the history of EGFR TKIs approval and emerging data. Cancers (Basel). (2023) 15. doi: 10.3390/cancers15030629 PMC9913773 36765587

[14] ten Berge DMHJ Damhuis RAM Aerts JGJV Dingemans A-MC. Real-world treatment patterns and survival of patients with ROS1 rearranged stage IV non-squamous NSCLC in the Netherlands. Lung Cancer. (2023) 181:107253. doi: 10.1016/j.lungcan.2023.107253 37236088

[15] Cancer Registry of Norway. Cancer in Norway 2022 - Cancer incidence, mortality, survival and prevalence in Norway. Oslo: Cancer Registry of Norway (2023).

[16] Kreftregisteret. Årsrapport 2018 med resultater og forbedringstiltak fra Nasjonalt kvalitetsregister for lungekreft. Oslo: Kreftregisteret (2019).

[17] INSPIRE:lungekreft. Evaluering av pilotprosjekt. Oslo: Kreftregisteret (2021).

[18] INSPIRE:lungekreft. Evaluering av pilotprosjekt: Vedlegg 9.1: Virkestoff og type behandling, tilordning/mapping. Oslo: Kreftregisteret (2021).

[19] Otto E Culakova E Meng S Zhang Z Xu H Mohile S. Overview of Sankey flow diagrams: Focusing on symptom trajectories in older adults with advanced cancer. J Geriatr Oncol. (2022) 13:742–6. doi: 10.1016/j.jgo.2021.12.017 PMC9232856 35000890

[20] Kaplan EL Meier P. Nonparametric estimation from incomplete observations. J Am Stat Assoc. (1958) 53:457–81. doi: 10.1080/01621459.1958.10501452

[21] Walker M Herms L Miller P. Performance of time to discontinuation and time to next treatment as proxy measures of progression-free survival, overall and by treatment group. J Clin Oncol. (2020) 38:e19135–5. doi: 10.1200/JCO.2020.38.15_suppl.e19135

[22] Wu Y-L Cheng Y Zhou X Lee KH Nakagawa K Niho S. Dacomitinib versus gefitinib as first-line treatment for patients with EGFR-mutation-positive non-small-cell lung cancer (ARCHER 1050): a randomised, open-label, phase 3 trial. Lancet Oncol. (2017) 18:1454–66. doi: 10.1016/S1470-2045(17)30608-3 28958502

[23] Soria J-C Ohe Y Vansteenkiste J Reungwetwattana T Chewaskulyong B Lee KH. Osimertinib in untreated EGFR-mutated advanced non-small-cell lung cancer. N Engl J Med. (2018) 378:113–25. doi: 10.1056/NEJMoa1713137 29151359

[24] Mok T Camidge DR Gadgeel SM Rosell R Dziadziuszko R Kim D-W. Updated overall survival and final progression-free survival data for patients with treatment-naive advanced ALK-positive non-small-cell lung cancer in the ALEX study. Ann Oncol. (2020) 31:1056–64. doi: 10.1016/j.annonc.2020.04.478 32418886

[25] Ahn MJ Kim HR Yang JHC Han J-Y Li JY-C Hochmair MJ. Efficacy and safety of brigatinib compared with crizotinib in Asian vs. Non-asian patients with locally advanced or metastatic ALK-inhibitor-naive ALK+ Non-small cell lung cancer: final results from the phase III ALTA-1L study. Clin Lung Cancer. (2022) 23:720–30. doi: 10.1016/j.cllc.2022.07.008 36038416

[26] Hizal M Bilgin B Paksoy N Kılıçkap S Atcı MM Kahraman S. Real-world data on efficacy and safety of first-line alectinib treatment in advanced-stage, ALK-positive non-small-cell lung cancer patients: a Turkish Oncology Group study. Future Oncol. (2022) 18:2573–82. doi: 10.2217/fon-2022-0083 35734870

[27] Gibson AJW Box A Dean ML Elegbede AA Hao D Sangha R. Retrospective real-world outcomes for patients with ALK-rearranged lung cancer receiving ALK receptor tyrosine kinase inhibitors. JTO Clin Res Rep. (2021) 2:100157. doi: 10.1016/j.jtocrr.2021.100157 34590010 PMC8474209

[28] Rupp M Fanton-Aita F Snow S Wheatley-Price P Melosky B Juergens RA. Lorlatinib effectiveness and quality-of-life in patients with ALK-positive NSCLC who had failed second-generation ALK inhibitors: Canadian real-world experience. Curr Oncol. (2023) 30:6559–74. doi: 10.3390/curroncol30070481 PMC10377946 37504341

[29] Shaw AT Riely GJ Bang Y-J Kim D-W Camidge DR Solomon BJ. Crizotinib in ROS1-rearranged advanced non-small-cell lung cancer (NSCLC): updated results, including overall survival, from PROFILE 1001. Ann Oncol. (2019) 30:1121–6. doi: 10.1093/annonc/mdz131 PMC6637370 30980071

[30] Mok TS Cheng Y Zhou X Lee KH Nakagawa K Niho S. Updated overall survival in a randomized study comparing dacomitinib with gefitinib as first-line treatment in patients with advanced non-small-cell lung cancer and EGFR-activating mutations. Drugs. (2021) 81:257–66. doi: 10.1007/s40265-020-01441-6 PMC7932969 33331989

[31] Su CC Wu JT Choi E Myall NJ Neal JW Kurian AW. Overall survival among patients with de novo stage IV metastatic and distant metastatic recurrent non–small cell lung cancer. JAMA Network Open. (2023) 6:e2335813–e2335813. doi: 10.1001/jamanetworkopen.2023.35813 37751203 PMC10523163

[32] Ramalingam SS Vansteenkiste J Planchard D Cho BC Gray JE Ohe Y. Overall survival with osimertinib in untreated, EGFR-mutated advanced NSCLC. N Engl J Med. (2020) 382:41–50. doi: 10.1056/NEJMoa1913662 31751012

[33] Wells JC Mullin MM Ho C Melosky B Laskin J Wang Y. Outcomes of patients with advanced epithelial growth factor receptor mutant lung cancer treated with first-line osimertinib who would not have met the eligibility criteria for the FLAURA clinical trial. Lung Cancer. (2024) 190:107529. doi: 10.1016/j.lungcan.2024.107529 38452600

[34] Nilssen Y Strand T-E Fjellbirkeland L Bartnes K Brustugun OT O'Connell DL. Lung cancer treatment is influenced by income, education, age and place of residence in a country with universal health coverage. Int J Cancer. (2016) 138:1350–60. doi: 10.1002/ijc.v138.6 26421593

[35] Eide IJZ Helland Å Ekman S Mellemgaard A Hansen KH Cicenas S. Osimertinib in T790M-positive and -negative patients with EGFR-mutated advanced non-small cell lung cancer (the TREM-study). Lung Cancer. (2020) 143:27–35. doi: 10.1016/j.lungcan.2020.03.009 32200138

[36] First-line treatment with osimertinib in EGFR-mutated Non-small cell lung cancer (FIOL) (2022). Available online at: https://clinicaltrials.gov/study/NCT03804580 (Accessed June 1, 2024).

[37] Eide IJZ Nilssen Y Stensland EM Brustugun OT. Real-world data on EGFR and ALK testing and TKI usage in Norway-A nation-wide population study. Cancers (Basel). (2023) 15. doi: 10.3390/cancers15051505 PMC10001166 36900294

[38] Kreftregisteret. Årsrapport 2017 med resultater og forbedringstiltak fra Nasjonalt kvalitetsregister for lungekreft. Oslo: Kreftregisteret (2018).

