# Corrigendum: Targeted treatment and survival in advanced non-squamous non-small cell lung cancer patients – a nationwide and longitudinal study

**DOI:** 10.3389/fonc.2025.1595028

**Published:** 2025-03-26

**Authors:** Johanne Elise Nyen, Anja Ødegård Booth, Øyvind Husby, Christoffer Bugge, Ingrid Engebretsen, Francisco Oteiza, Åslaug Helland, Lars Fjellbirkeland, Odd Terje Brustugun, Bjørn Henning Grønberg

**Affiliations:** ^1^ Oslo Economics, Oslo, Norway; ^2^ Pfizer AS, Oslo, Norway; ^3^ Institute of Clinical Medicine, University of Oslo, Oslo, Norway; ^4^ Division of Cancer Medicine, Oslo University Hospital, Oslo, Norway; ^5^ Department of Respiratory Medicine, Oslo University Hospital, Oslo, Norway; ^6^ Section of Oncology, Drammen Hospital, Vestre Viken Hospital Trust, Drammen, Norway; ^7^ Department of Clinical and Molecular Medicine, Norwegian University of Science and Technology (NTNU), Trondheim, Norway; ^8^ Department of Oncology, St. Olavs Hospital, Trondheim University Hospital, Trondheim, Norway

**Keywords:** ROS, ALK, EGFR, real world data, lung cancer, time on treatment

In the published article, there was an error. The ClinicalTrials.gov-identifier of our study was not included in the publication.

A correction has been made to **Materials and methods**, *Reporting guidelines and ethics*, Paragraph 1. This sentence previously stated:

“The study was approved by the Regional Ethics Committee of Norway South-East D (Reference number 485084)”.

The corrected sentence appears below:

“The study was approved by the Regional Ethics Committee of Norway South-East D (Reference number 485084) and registered at http://ClinicalTrials.gov (Reference number NCT05834348)”.

The authors apologize for this error and state that this does not change the scientific conclusions of the article in any way. The original article has been updated.

