# Supplementary material for: Targeted treatment and survival in advanced non-squamous non-small cell lung cancer patients – a nationwide and longitudinal study
Source: Front Oncol. 2025 Feb 20;15:1506041. doi: 10.3389/fonc.2025.1506041 (PMC11882418; doi:10.3389/fonc.2025.1506041)
Supplement: Supplementary file 1 [file DataSheet1.docx]

Supplementary Material

Supplementary figure 1: Time on second line treatment

| A: Time on second line treatment post osimertinib in first line  (EGFR+) |  |  |  | B: Time on lorlatinib in second line after alectinib (ALK+) | |
| --- | --- | --- | --- | --- | --- |
| 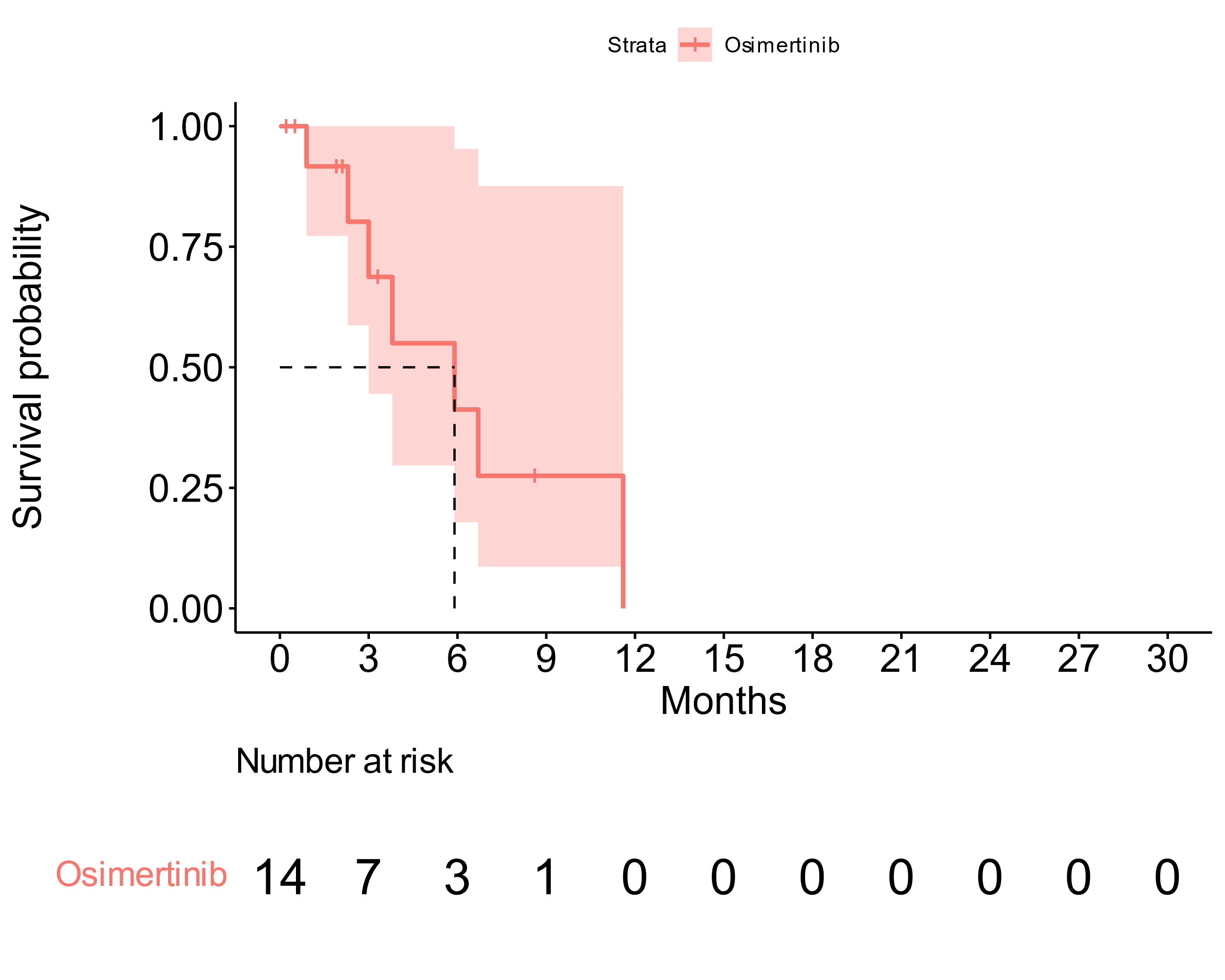 |  |  |  |  | 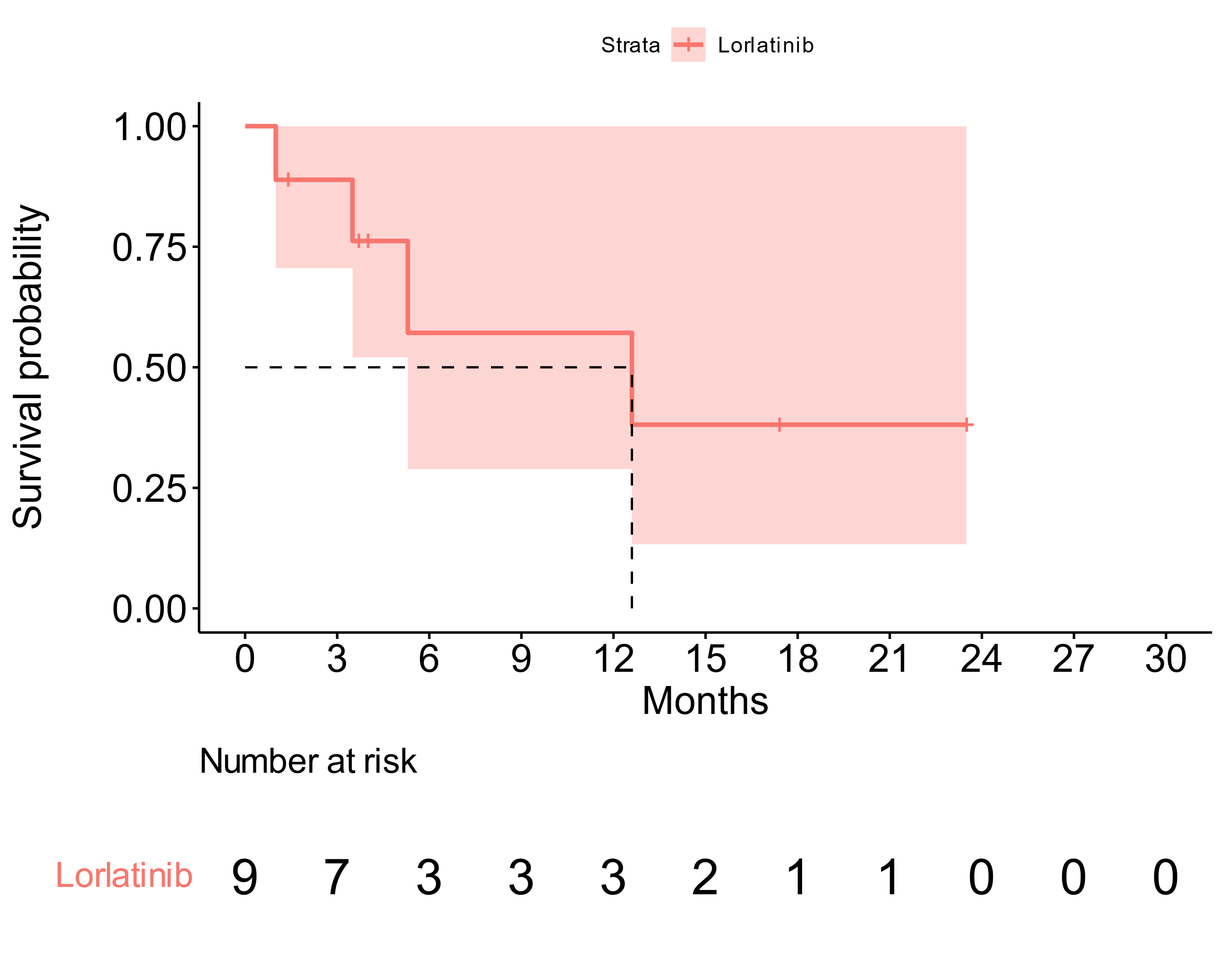 |
| **mToT (95% CI)**  Second line post osimertinib: 5.9 months (3.0-NA) |  |  |  |  | **mToT (95% CI)**  Second line lorlatinib: 12.6 months (5.3-NA) |

Note: Kaplan Meier plots showing time on second line treatment for patients in EGFR subgroup treated with osimertinib (A) and patients in the ALK subgroup treated with lorlatinib after alectinib (B). Patients who received treatment post Osimertinib received quadruple treatment (N=10), platinum doublet (N=1), gefitinib (N=1), dacomitinib (N=1), and immunotherapy (N=1). CI = confidence interval.

Supplementary figure 2: Time on treatment after targeted treatment

| A: Time on platinum doublet or quadruple treatment after targeted therapy in EGFR subgroup |  |
| --- | --- |
| 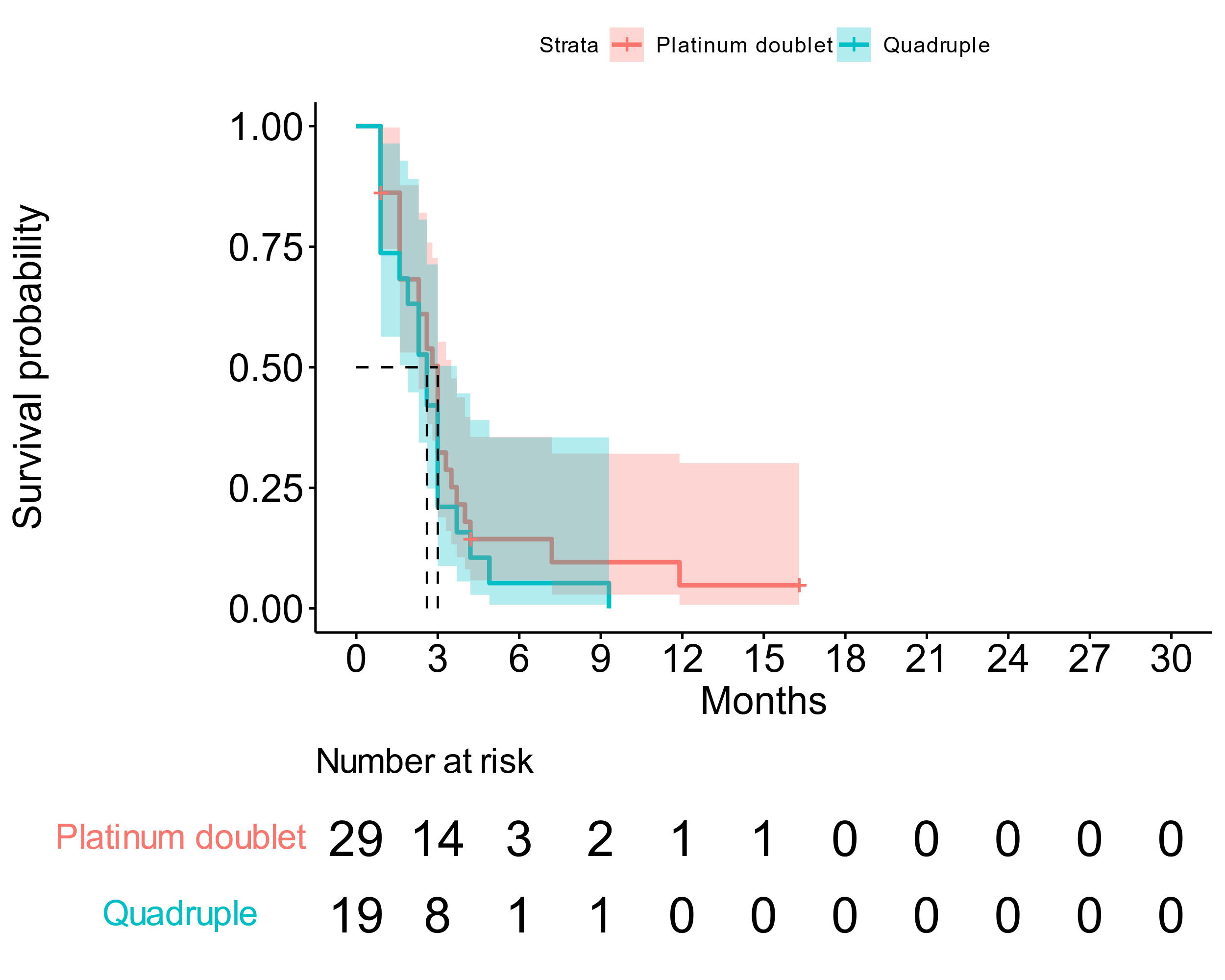 |  |
| **mToT (95% CI)**  Platinum doublet: 3.0 months (2.3-3.5)  Quadruple: 2.6 months (1.9-3.7) |  |

Note: Kaplan Meier plots showing time on quadruple and platinum doublet treatment for patients in EGFR subgroup. Only individuals who receive the platinum doublet or quadruple treatment after receiving targeted treatment are included in the analysis. Quadruple treatment refers to atezolizumab, bevacizumab, paclitaxel and carboplatin administered within the same week. Platinum doublet refers to treatment with cisplatin or carboplatin in combination with vinorelbine, etoposide, paclitaxel, pemetrexed or gemcitabine administered within the same week. CI = confidence interval.

Supplementary table 1: First line targeted treatment among EGFR patients, by year of diagnosis

|  | **2015** | **2016** | **2017** | **2018** | **2019** | **2020** | **2021** | **2022** |
| --- | --- | --- | --- | --- | --- | --- | --- | --- |
| **Afatinib** | <5 | 5 | 5 | <5 |  | <5 |  | <5 |
| **Erlotinib** | 7 | 20 | 22 | <5 |  |  |  |  |
| **Gefitinib** | 5 | <5 | 10 | 36 | 22 | 9 | <5 |  |
| **Crizotinib** |  |  |  | <5 |  | <5 |  |  |
| **Dacomitinib** |  |  |  |  | <5 | 24 | <5 |  |
| **Osimertinib** |  |  |  |  |  |  | 43 | 61 |
| **Entrectinib** |  |  |  |  |  |  |  | <5 |

Note: Table shows number of EGFR+-patients receiving each targeted therapy as first line treatment by year of diagnosis.

Supplementary table 2: First line targeted treatment among ALK patients, by year of diagnosis

|  | **2015** | **2016** | **2017** | **2018** | **2019** | **2020** | **2021** | **2022** |
| --- | --- | --- | --- | --- | --- | --- | --- | --- |
| **Crizotinib** |  | 8 | 13 | 7 |  | <5 |  |  |
| **Alectinib** |  |  |  | 6 | 19 | 15 | 15 |  |
| **Brigatinib** |  |  |  |  |  |  | 8 | 13 |

Note: Table shows number of ALK+-patients receiving each targeted therapy as first line treatment by year of diagnosis.

Supplementary table 3: One and two-year survival, by subgroup

|  | **All biomarker subgroups** | **Other Non-Squamous NSCLC** | **EGFR +** | **ALK +** |
| --- | --- | --- | --- | --- |
| **First period** | **2015-2019** | **2015-2019** | **2015-2019** | **2016-2019** |
| 1-year OS (95% CI) | 64.1 %  (58.9-69.8 %) | 28.2 %  (26.6-30.0 %) | 62.4 %  (56.5-69.0 %) | 69.1 %  (59.0-81.0 %) |
| 2-year OS (95% CI) | 39.9 %  (34.7-45.9 %) | 15.5 %  (14.2-17.0 %) | 37.1 %  (31.4-43.9 %) | 48.5 %  (38.0-62.0 %) |
| **Second period** | **2020-2022** | **2020-2022** | **2020-2022** | **2020-2022** |
| 1-year OS (95% CI) | 70.2 %  (64.9-76.0 %) | 34.6 %  (32.4-37.0 %) | 71.3 %  (65.0-78.2 %) | 77.7 %  (67.6-89.3 %) |
| 2-year OS (95% CI) | 49.7 %  (42.7-57.9 %) | 22.4 %  (20.2-24,9 %) | 47.6 %  (39.3-57.5 %) | 62.6 %  (48.4-81.1 %) |

Supplementary figure 3: Time on treatment on each targeted therapy in first line for EGFR patients

| A: Time on erlotinib or gefitinib as first line treatment | B: Total treatment time (all lines combined) when starting on erlotinib or gefitinib | |
| --- | --- | --- |
| 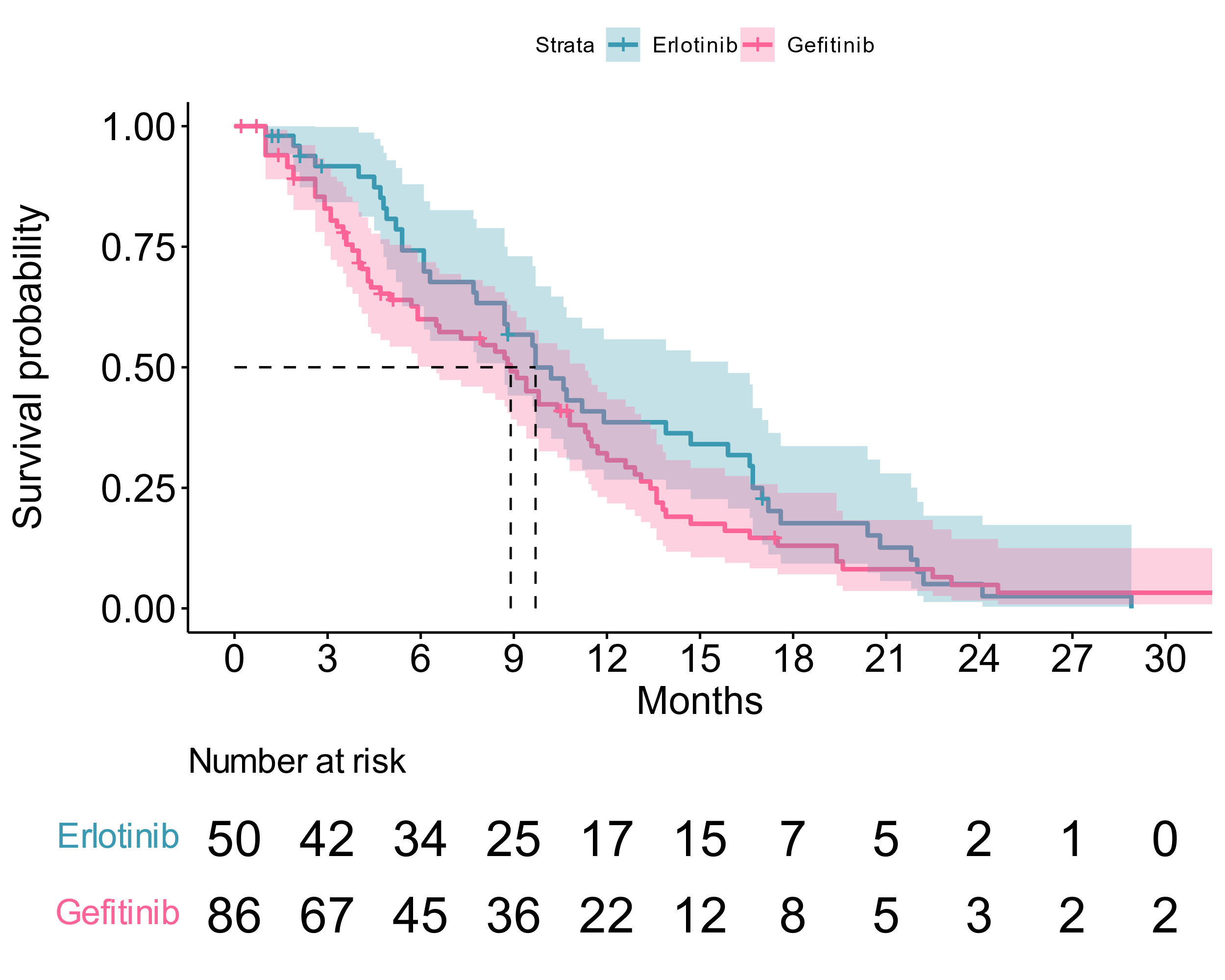 |  | 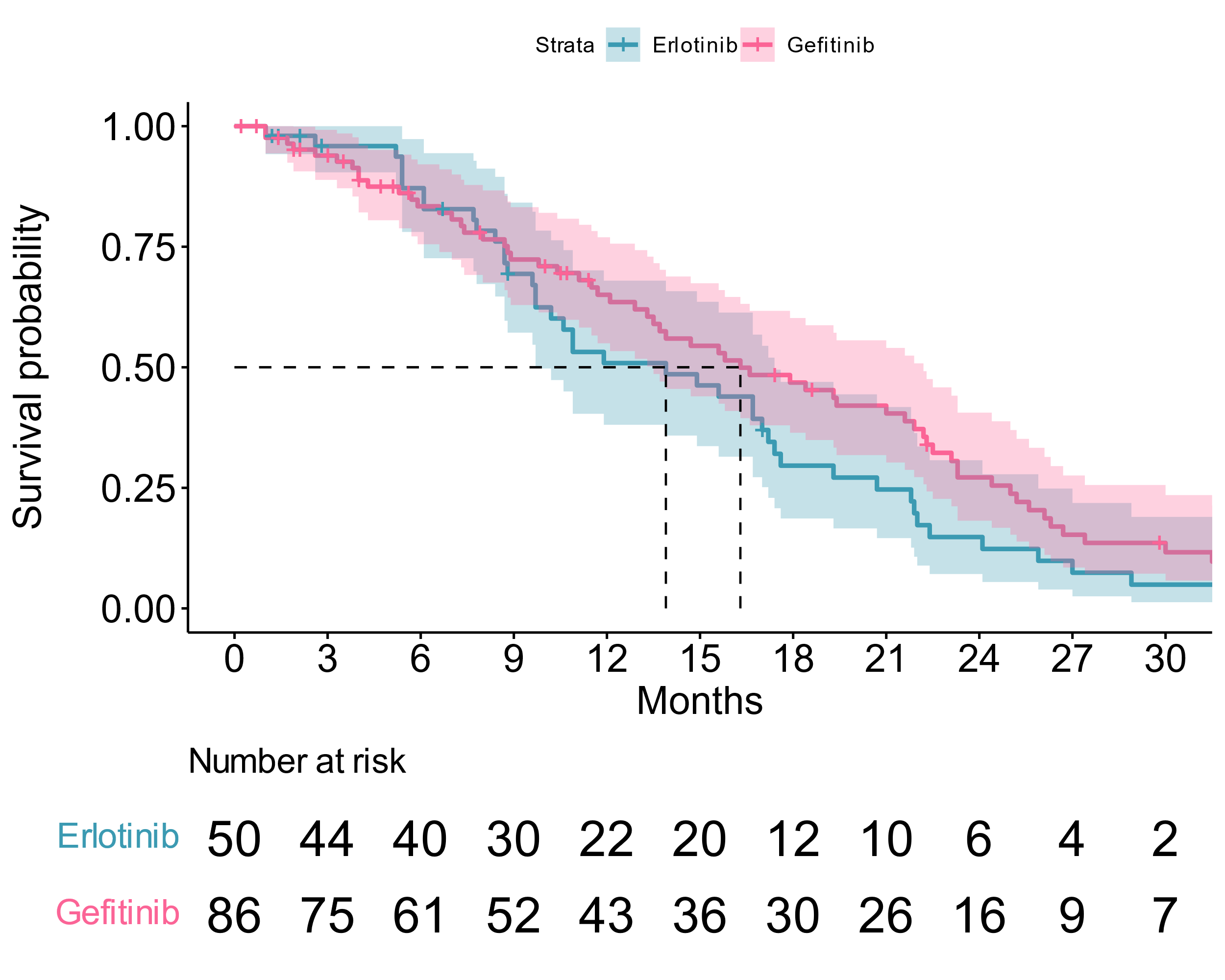 |
| **mToT (95% CI)** Erlotinib: 9.7 months (8.7-15.9)  Gefitinib = 8.9 months (6.5-11.3) |  | **mTToT (95% CI)**  Erlotinib = 13.9 months (9.7-13.5)  Gefitinib = 16.3 months (13.5-22.2) |
| **C: Time on afatinib, dacomitinib or osimertinib as first line treatment** |  | **D: Total treatment time (all lines combined) when starting on afatinib, dacomitinib or osimertinib** |
| 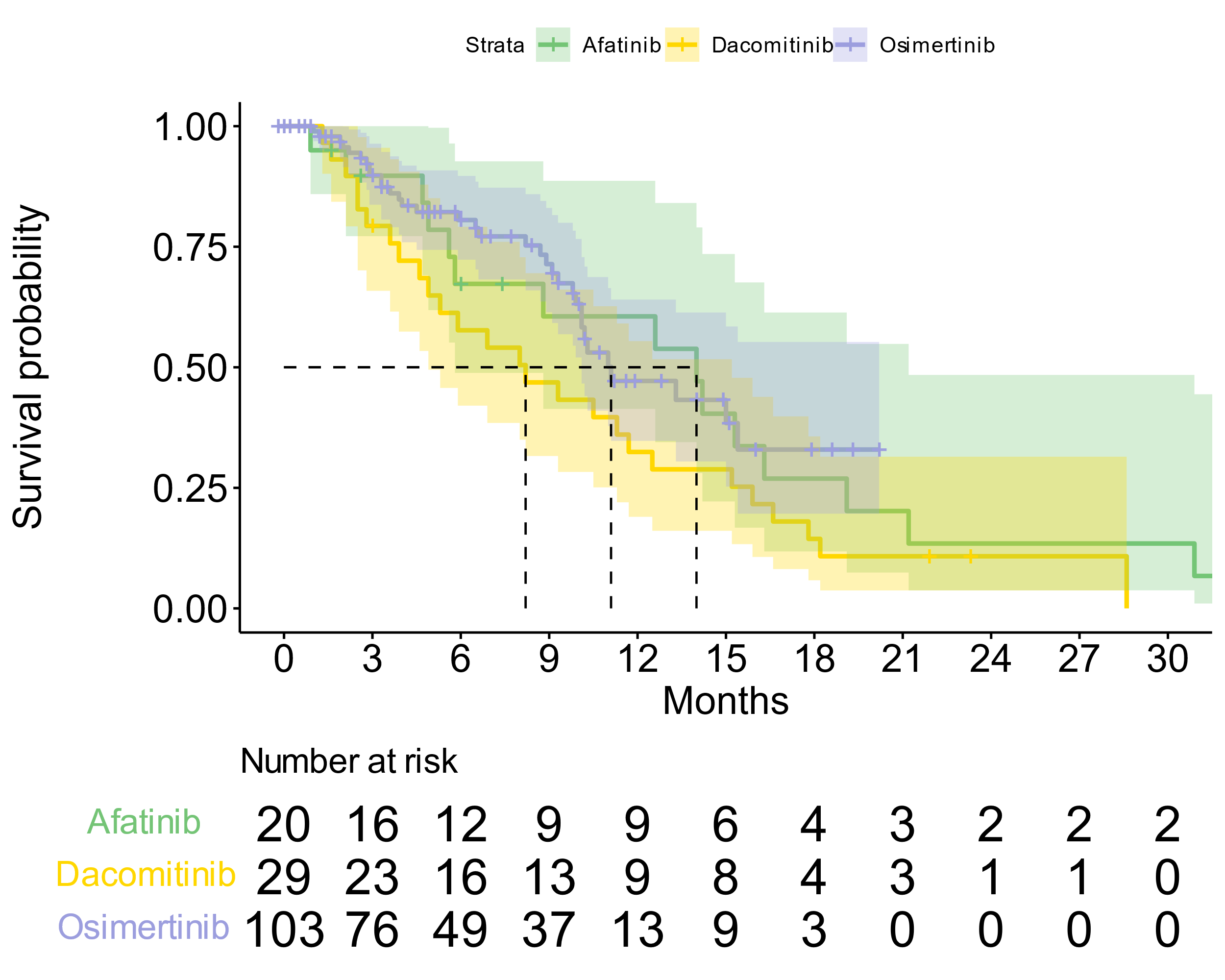 |  | 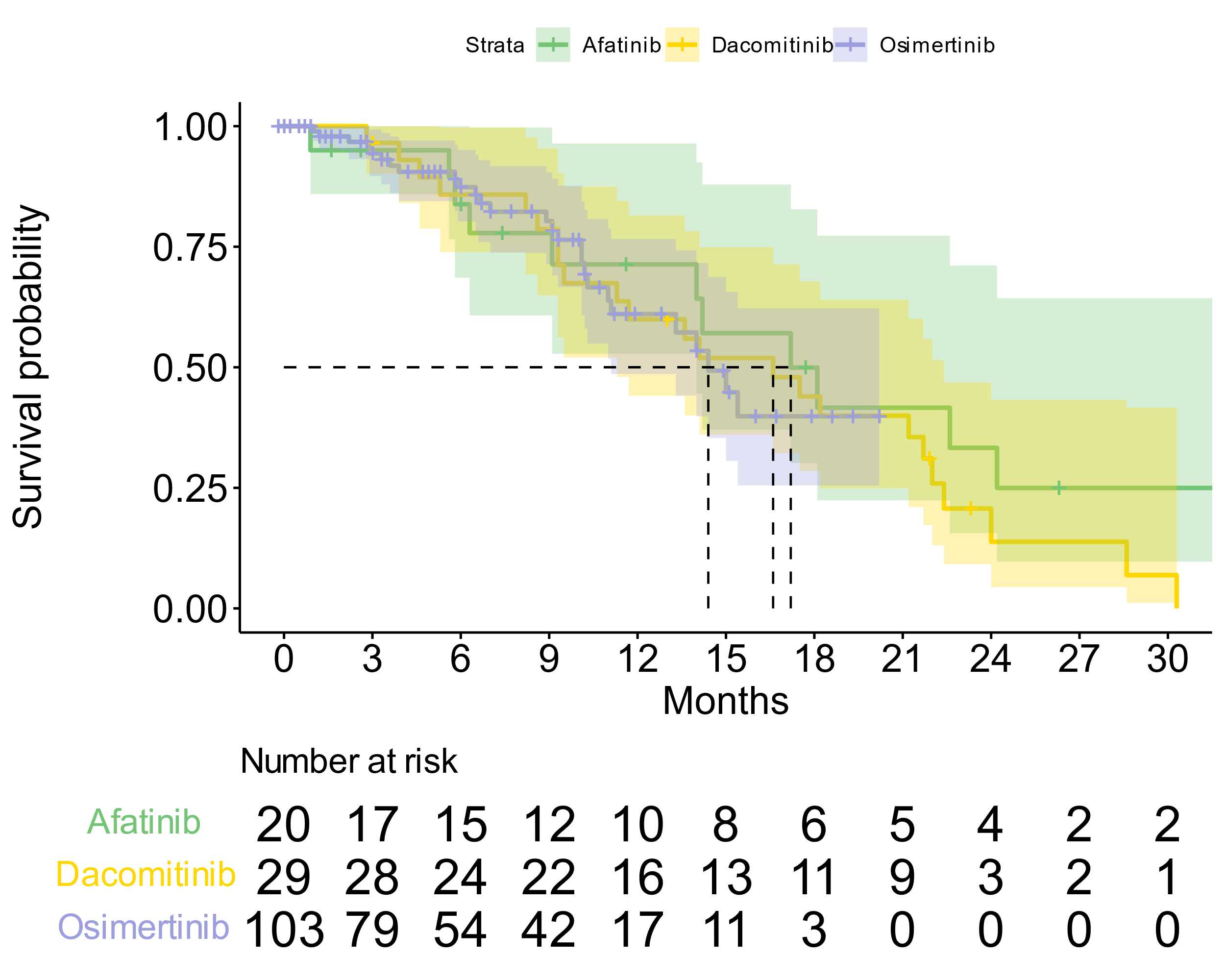 |
| **mToT (95% CI)**  Afatinib = 14 months (5.8-21.2)  Dacomitinib = 8.2 months (4.9-15.2)  Osimertinib: 11.1 months (10.1-NA) |  | **mTToT (95% CI)**  Afatinib = 17.2 months (14.0-NA)  Dacomitinib = 16.6 months (11.3-22.4)  Osimertinib =14.4 months (11.1-NA) |

CI = confidence interval.

Supplementary figure 4: Length of treatment duration for each patient (Swimmer plots)

| A1: EGFR+ (patients treated with erlotinib, dacomitinib or osimertinib in first line) |  |
| --- | --- |
| 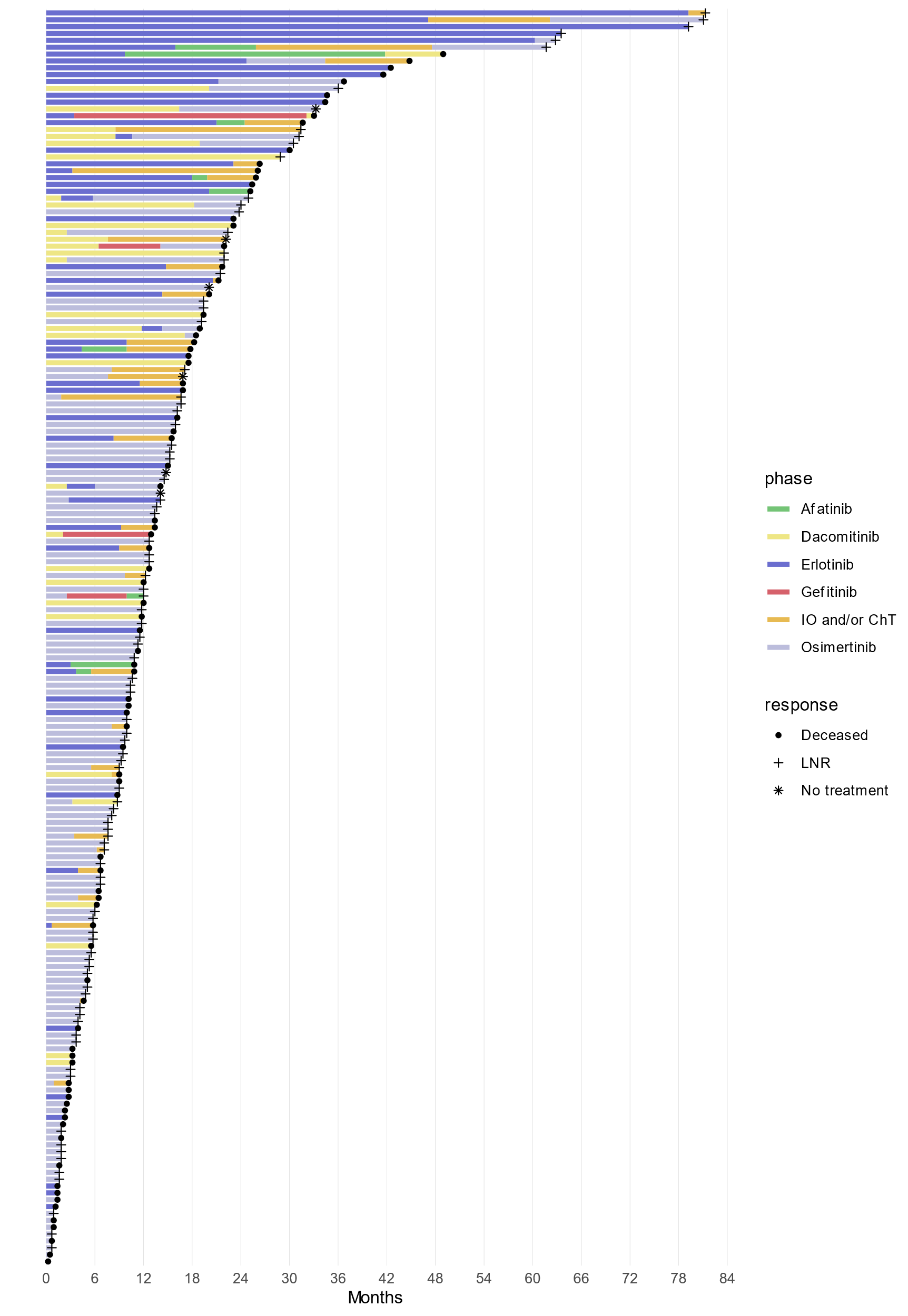 |  |
| **A2: EGFR+ (patients treated with other treatments in first line)** |  |
| 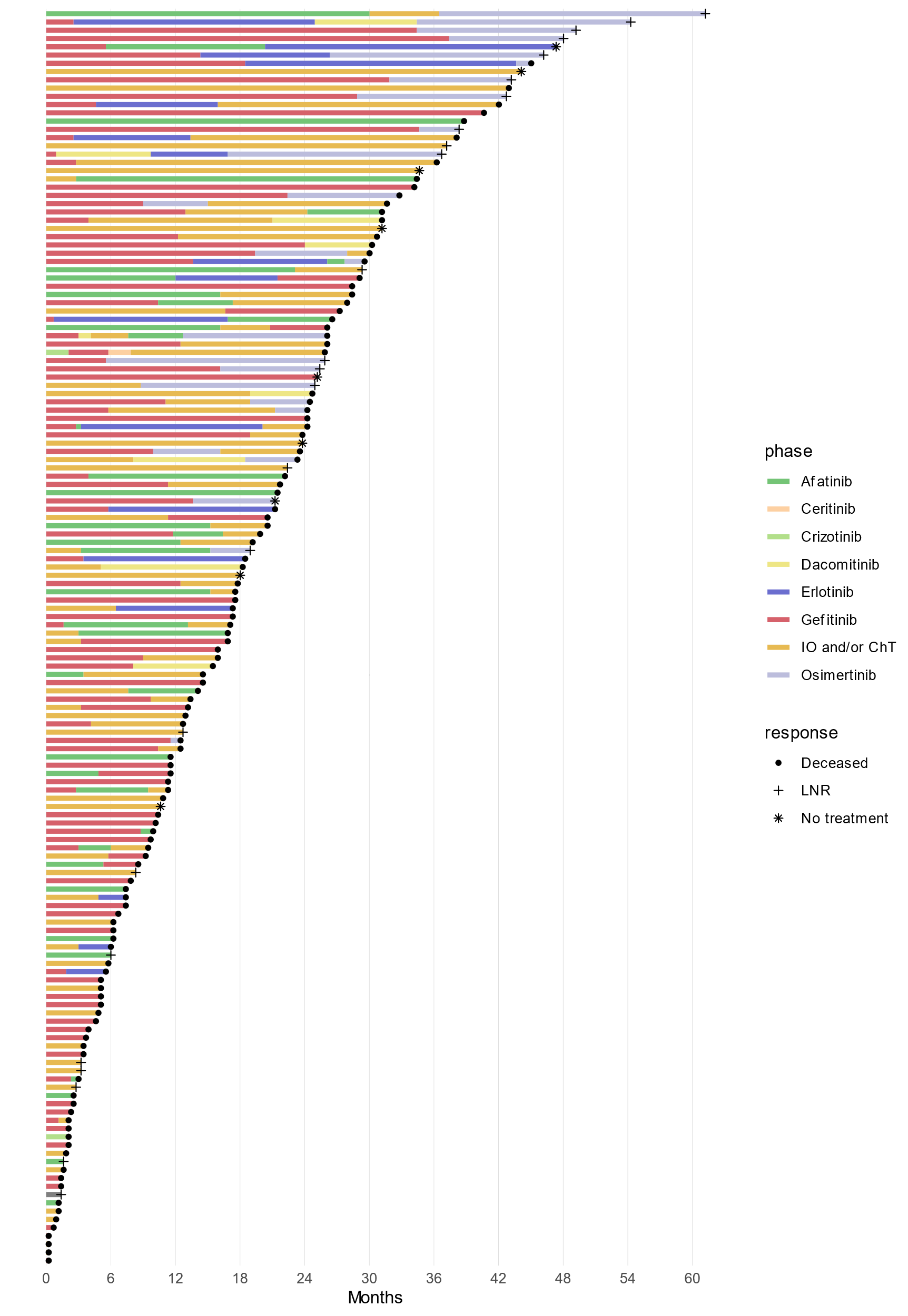 |  |
| **B: ALK+** |  |
| 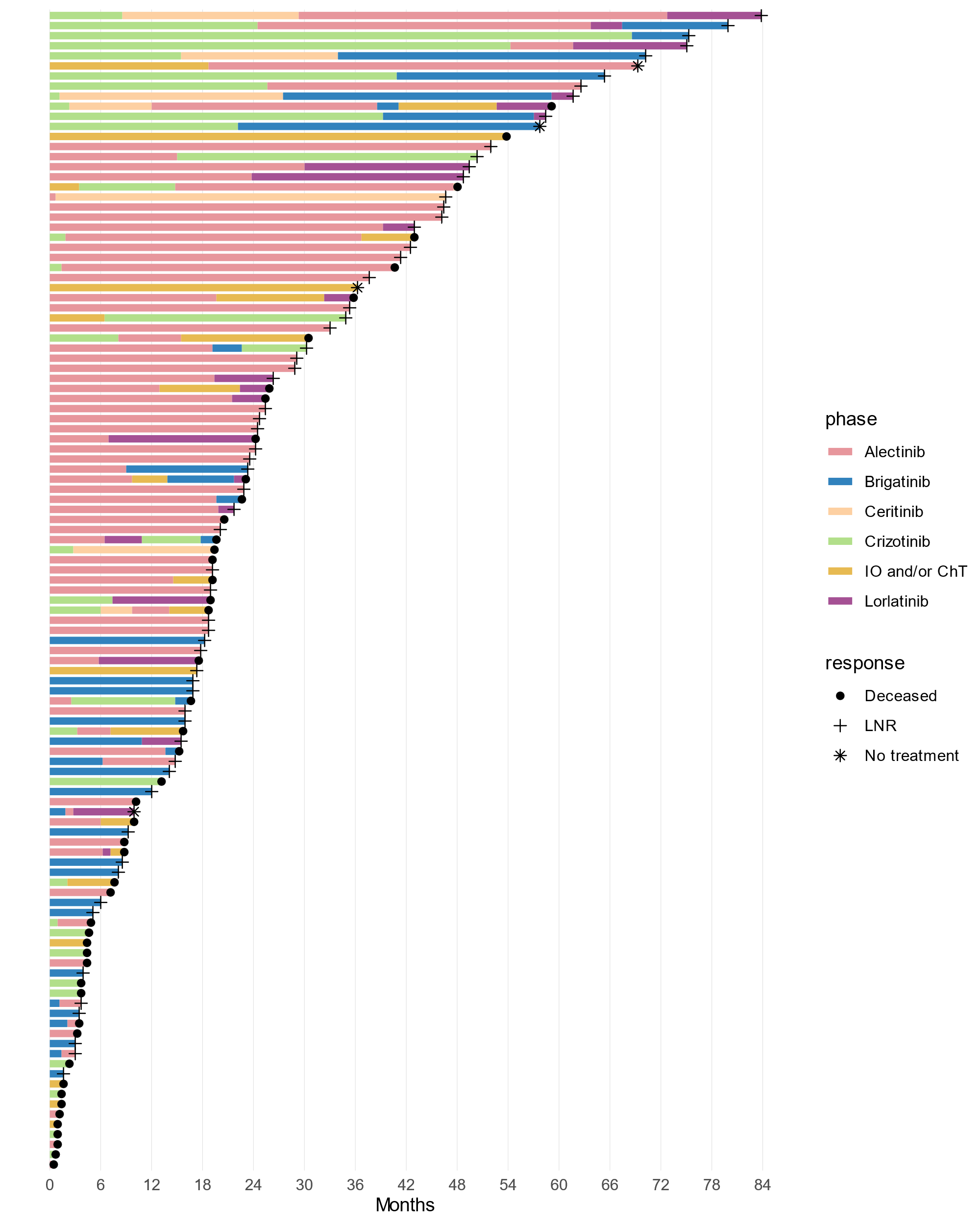 |  |
|  |  |
|  |  |
|  |  |
| **C: ROS1+** |  |
| 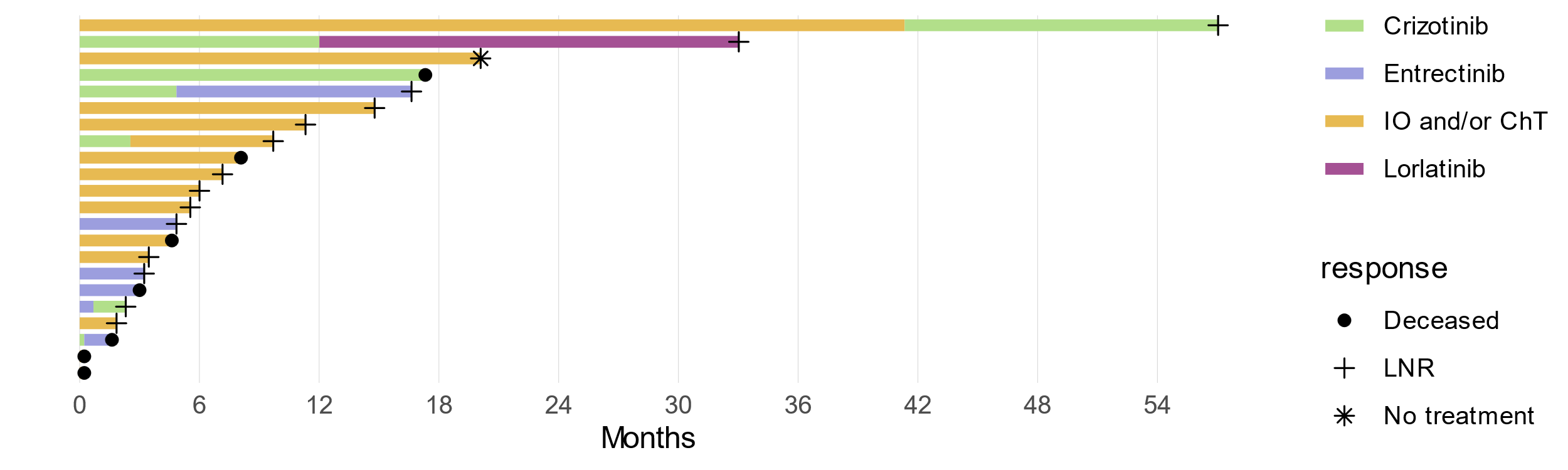 |  |
